# Antimicrobial Resistance of Non-Fermenting Gram-Negative Bacilli in a Multidisciplinary Hospital in Romania

**DOI:** 10.3390/biomedicines13092255

**Published:** 2025-09-12

**Authors:** Miruna-Maria Apetroaei, Mihaela Cristina Negulescu, Sorina Hîncu, Adriana Tăerel, Manuela Ghica, Andreea Letiția Arsene, Denisa Ioana Udeanu

**Affiliations:** 1 Faculty of Pharmacy, Carol Davila University of Medicine and Pharmacy, 6, Traian Vuia Street, 020956 Bucharest, Romania; miruna-maria.apetroaei@rez.umfcd.ro (M.-M.A.); sorina.calugaru@drd.umfcd.ro (S.H.); manuela.ghica@umfcd.ro (M.G.); andreea.arsene@umfcd.ro (A.L.A.); denisa.udeanu@umfcd.ro (D.I.U.); 2 Faculty of Medicine, Carol Davila University of Medicine and Pharmacy, 8, Eroii Sanitari Street, 050474 Bucharest, Romania; mihaela-cristina.bondoc@drd.umfcd.ro; 3 Fundeni Clinical Institute, 258, Fundeni Street, 022328 Bucharest, Romania; 4 Marius Nasta Institute of Pneumonology, 90, Viilor Street, 050159 Bucharest, Romania

**Keywords:** antimicrobial resistance, MARI, Gram-negative bacilli, nosocomial infections, multidrug-resistant pathogens, antimicrobial stewardship

## Abstract

**Background:** Antimicrobial resistance (AMR) in *Acinetobacter* spp., *Pseudomonas* spp., and *Stenotrophomonas maltophilia* poses a significant risk in healthcare-associated infections. Constant monitoring using quantitative metrics is necessary to direct empirical treatment. **Methods:** We conducted a retrospective observational study at the Fundeni Clinical Institute, Bucharest, Romania, analysing antibiogram data from January 2021 to December 2024. Over 200,000 microbiological records were screened, and 1189 isolates of the three targeted pathogens were included. The Multiple Antibiotic Resistance Index (MARI) was applied to evaluate selective pressure across years, hospital departments, sample types, and hospitalisation categories. **Results:** *Acinetobacter baumannii* and *Pseudomonas aeruginosa* exhibited the highest resistance levels, with median MARI values exceeding 0.25 in 2024, particularly in Intensive Care and Transplant units. In contrast, *S. maltophilia* showed lower overall MARI values, though resistance variability increased in 2024 (extremes up to 0.30). Notably, resistance to carbapenems in *Acinetobacter* spp. rebounded in 2024, while *Pseudomonas* spp. demonstrated a favourable trend of decreasing resistance to several β-lactams. **Conclusion:** Our findings underscore significant interspecies differences in AMR dynamics and highlight the utility of MARI as a valuable operational indicator. Ongoing local surveillance is needed for refining empirical treatment protocols and informing antimicrobial stewardship in Romanian hospitals.

## 1. Introduction

Antimicrobial resistance (AMR) is a significant public health issue of the contemporary era, with an estimated 10 million deaths annually by 2050. The primary cause of this phenomenon is the widespread misuse of antibiotics, which results in the selection of bacteria that are resistant to them. The resulting infections are more challenging to treat, leading to severe illness and an increased risk of disease dissemination and mortality [1,2].

The substantial public health hazard that bacterial AMR poses to healthcare systems, economies, and communities is underscored by recent estimates of fatalities caused by AMR [3]. Hospital costs, utility loss, and the risk of death per patient may be elevated due to antibiotic resistance [4]. It is imperative to fully understand these losses on a national, regional, and global scale in order to address AMR effectively. The economic burden is linked to a large number of hospital bed-days occupied, healthcare expenditure, and labour productivity losses on a global scale. Consequently, it should remain a high priority on national and international policy initiatives [5]. Global modelling studies generate significant statistics that serve to motivate and direct global and national agendas. However, their complex methodology and aggregation result in a more restricted impact at the local level, where AMR is experienced and addressed [3].

WHO Member States strongly endorsed a Global Action Plan to combat AMR in 2015, aiming to maintain efficient prevention and management of infectious diseases with effective, safe, quality-assured, responsible, and accessible medications for as long as feasible. Policy and disease prevention and control decisions depend on surveillance. Therefore, it is of utmost importance to measure the spread of AMR and monitor local, national, and global initiatives. On October 22, 2015, the WHO launched the worldwide Global Antimicrobial Resistance and Use Surveillance System (GLASS), the first worldwide shared AMR monitoring initiative [6]. Moreover, One Health encompasses a range of initiatives aimed at establishing enforcement programs, laws, policies, and research that facilitate collaboration across various sectors to improve public health outcomes. In order to mitigate public health hazards at the interface of the environment, humans, and animals, this approach is required [7,8]. To effectively address AMR, it is recommended to adopt a One Health integrated approach that encompasses the health sectors of animals, humans, and the environment. Finally, the Sustainable Development Goals must be achieved by addressing AMR through One Health, which emphasises the importance of fostering partnerships across different sectors [9,10].

AMR surveillance involves the systematic gathering, analysis, and interpretation of data regarding the occurrence and distribution of antimicrobial resistance in humans, animals, and the environment [11]. This analysis offers insights into trends, patterns, and factors related to AMR, facilitating evidence-based decision-making for the implementation of suitable measures [12].

In June 2018, a joint team by the European Commission, ECDC, and WHO visited Romania to assess the national situation regarding AMR and antimicrobial consumption. The main conclusions were that Romania faced very high rates of antimicrobial resistance, particularly among Gram-negative pathogens, combined with high levels of antibiotic consumption and gaps in infection control. The recommended actions included developing and implementing a national AMR strategy, reinforcing antimicrobial stewardship in hospitals, improving surveillance systems for both AMR and antibiotic use, and investing in infection prevention and control programs [13]. Additionally, in 2019, the European Public Health Alliance (EPHA) published the report “In the Red Zone”, which highlighted the severity of AMR in Romania compared to other European Union countries. The document emphasised alarmingly high levels of resistance in Gram-negative pathogens and underscored weaknesses in national surveillance, stewardship, and infection prevention strategies [14]. In this direction, Blejan et al. examined 170 cases of community-acquired pneumonia (CAP) at the Bucharest Clinical Emergency Hospital from December 2017 to 2018. *Klebsiella* sp., *Acinetobacter baumannii*, *Staphylococcus aureus*, *Escherichia coli*, and *Pseudomonas* sp. were the most prevalent bacteria found in refractory antibiotic patients. *A. baumannii* and *Klebsiella* sp., which were resistant to carbapenems and fluoroquinolones, had high Multiple Antibiotic Resistance Index (MARI)values. The study concluded that resistant and multidrug-resistant bacteria affect Romanian CAP outcomes, emphasising the need for national surveillance and antibiotic management [15].

Unfortunately, there have been numerous reports that the onset of the COVID-19 pandemic in Romania has exacerbated this situation [16,17]. In the same direction, to evaluate bacterial infections, trends, and resistance patterns during the COVID-19 pandemic in Romania, Vulcanescu et al. conducted a comprehensive systematic review. Included were 87 papers that examined more than 20,000 bacterial infections. Gram-negative bacteria, including *Escherichia coli* and *Klebsiella pneumoniae*, as well as Gram-positive bacteria such as *Staphylococcus aureus* and *Enterococcus* species, were the most commonly identified pathogens. Common resistance to carbapenems and cephalosporins was observed in 24% of the organisms reported to have MDR. The authors concluded that treating bacterial infections has become more difficult as a result of the pandemic, especially for patients in severe condition [18].

Although there are multiple warnings about the danger posed by increased antibiotic resistance in various regions of Romania, surveillance measures remain insufficient, and data are underreported. The AMR situation in Romania requires continuous reporting of infections and stewardship measures. Consequently, a recent multidisciplinary hospital-based study from North-Eastern Romania reported that the most prevalent healthcare-associated infections were respiratory tract and urinary tract infections, followed by surgical wound infections and sepsis, with the highest burden in intensive care units, surgical departments, and medical wards. The predominant pathogens isolated were *Acinetobacter baumannii*, *Klebsiella pneumoniae*, and *Pseudomonas aeruginosa*, alongside *Escherichia coli* and *Staphylococcus aureus*, illustrating the high prevalence of multidrug-resistant Gram-negative bacteria in Romanian hospital settings [19].

The capacity to adjust infection control and antimicrobial stewardship programs to local trends is restricted by the lack of and frequent underreporting of regional data on AMR in Romania. This investigation aimed to assess the AMR profiles of *Acinetobacter* spp., *Pseudomonas* spp., and *Stenotrophomonas maltophilia* in a national reference hospital in Bucharest over four years by using a quantitative index, MARI. The novelty of our work relies on the longitudinal, hospital-level data we have collected, focusing on the most prevalent Gram-negative bacteria that have been previously identified in other Romanian regions. Additionally, we have included *S. maltophilia*, an emerging pathogen that is frequently underreported, despite its clinical impact and limited therapeutic options. Our study aims to address the lack of reliable local surveillance data that could guide empirical treatments and care policies in Romania.

## 2. Materials and Methods

### 2.1. Study Design

This retrospective, observational study evaluated the antimicrobial resistance profile of nosocomial pathogens isolated from clinical samples collected during routine clinical practice at the Fundeni Clinical Institute, a multidisciplinary hospital in Bucharest, Romania. The research was conducted over a period of four consecutive years, from 1 January 2021, to 31 December 2024, without seasonal or institutional interruptions, and reflects the current activity of the microbiology laboratory at this medical institution. Microbiological testing is integrated into the clinical workflow of diagnosis and therapeutic monitoring, with samples being collected at the attending physician’s request and processed in a standardised manner and results being reported electronically within the information system. The data analysed were extracted from the Microbiology Laboratory database and included all microbiological test records performed during the study period.

As the study was based exclusively on anonymised data obtained from electronic laboratory files, without involving additional interactions with patients or access to sensitive medical information, it falls within the category of studies with minimal ethical risk. Although patient consent was not required for the retrospective use of data, the processing of information was carried out in accordance with the principles regulated by the legislation in force on the protection of personal data (Regulation (EU) 2016/679-GDPR and approved by the Ethics Committee of the Fundeni Clinical Institute (No. 11173/18 March 2025)

The study was designed to allow comparability between years, between departments, and between types of hospitalisation and types of biological products. Therefore, a standardised procedure was applied for selecting records and defining clinical and microbiological variables. The variables of interest were then coded for statistical analysis so that significant differences between groups could be investigated and longitudinal trends in antimicrobial resistance levels at the institution studied could be estimated.

### 2.2. Data Analysis

The initial database included all records related to biological samples analysed during the study period, regardless of the pathogen identified or the type of antibiogram performed. The file was structured as a table, containing the following categories: department/ward of origin; type of hospitalisation (inpatient or outpatient); observation record code, type of analysis requested (urine culture, blood culture, secretions, etc.); isolated microorganism; antibiogram result, in a single cell in the form of a text string containing all the antibiotics tested and the associated interpretations; and date of request and date of reporting of the result, in some cases expressed in multiple fields (day, month, year).

To ensure compliance with ethical requirements and personal data protection rules, all identifiable information was removed during processing. Additionally, cases with incomplete records, those lacking microbiological information, or instances where the antibiogram was unavailable were excluded.

Furthermore, for each record, the list of antibiotics was fragmented, and each antibiotic tested was transferred to a separate column in a categorical format, such as “R” for resistant, “S” for susceptible, “I” for intermediate [20], and “N” for not tested/absent.

Thus, the final database used in the analysis included the following variables: year of isolation, patient’s department of origin, type of hospitalisation, type of biological sample, identified microorganism, and antibiogram results.

### 2.3. Selected Microorganisms, Sample Types, and Inclusion/Exclusion Criteria

For this analysis, three bacterial species considered representative of severe nosocomial infections and characterised by an increased potential for developing multidrug resistance (MDR) were selected: *Acinetobacter* spp., *Pseudomonas* spp., *and Stenotrophomonas maltophilia* [21]. These were selected based on the following considerations: (1) high prevalence in intensive care units and other high-risk wards in the hospital analysed [22,23]; (2) intrinsic ability to develop multiple resistance to commonly used antibiotics, including carbapenems, fluoroquinolones, aminoglycosides, and β-lactam/β-lactamase inhibitor combinations [21]; (3) clinical relevance in empirical treatments and current guidelines for the management of severe infections; (4) strategic interest in their surveillance, in the context of the priorities defined by the WHO for critical pathogens for which new antibiotics are needed [24].

After the selection of the three microorganisms, only cases in which they were identified in biological samples of diagnostic interest were extracted from the initial database, and the following types of samples were included in the analysis: bronchial secretions (relevant in lower respiratory tract infections, especially in mechanically ventilated patients or patients with nosocomial bronchopneumonia), sputum (used to assess respiratory infections), urine culture (useful in documenting complicated urinary tract infections, with medical indication for microbiological testing and interpretation of the clinical significance of the isolated strain), and blood culture (standard in the diagnosis of bacteraemia and sepsis, collected either peripherally or through a central catheter, interpreted according to the laboratory’s internal protocol).

Finally, the inclusion criteria for this analysis were as follows: (1) identification of a bacterial strain belonging to one of the three selected species; (2) existence of a complete antibiogram with clear interpretation for a sufficient number of antibiotics (≥5 molecules tested); (3) origin of the sample from one of the four types of biological products considered relevant; (4) complete recording of the clinical variables necessary for the analysis (year, department, type of hospitalisation, type of sample, tested antibiotics); (5) cases of mono-infection, without association with other bacterial strains in the same sample. The exclusion criteria also included the following: (1) samples in which polymicrobial cultures were identified; (2) results described as “mixed flora”, “possible contaminant”, or without clear clinical relevance; (3) samples from superficial biological sources (pharyngeal exudate, nasal secretions, wound secretions without indication of local sepsis); (4) cases lacking variables for statistical analysis (lack of interpretation of the antibiogram, lack of coding of the type of hospitalisation or ward).

### 2.4. Bacterial Identification and Antibiograms

The bacterial identification and antimicrobial susceptibility testing process was performed in the hospital’s microbiology laboratory, according to standard working procedures applied in routine clinical practice. Microorganisms were identified using automated methods. The interpretation of the antibiogram results was performed in accordance with the guidelines developed by the European Committee on Antimicrobial Susceptibility Testing (EUCAST) in force at the time of testing. The records were coded in a standardised format, with each antibiotic classified into one of three categories: susceptible, susceptible at increased exposure, and resistant.

The number and type of antibiotics tested varied depending on the isolated species, hospital-specific protocols, and the availability of test kits in the years analysed. Additionally, in some cases, certain molecules were replaced with pharmacologically equivalent alternatives or omitted from the antibiogram if they were not relevant to the specific infection.

### 2.5. Calculation of the Multiple Antibiotic Resistance Index

The assessment of the degree of antimicrobial resistance of each isolated bacterial strain was performed by applying a standardised quantitative indicator, Multiple Antibiotic Resistance Index (MARI). This index allows the proportion of antibiotics to which a strain is classified as resistant to be quantified, providing a summary measure of selective pressure and potential prior exposure to antimicrobials [25]. The formula used to calculate the MAR was

MARI = a/b(1)

where a is the number of antibiotics for which the strain was classified as R; b represents the total number of antibiotics tested for that strain.

Each MARI value was calculated individually, per record line, after the database had been completely processed.

According to criteria accepted in the literature, a MARI ≥ 0.2 was considered suggestive of strains originating from sources with increased exposure to antibiotics, i.e., from a clinical setting with high antimicrobial pressure [26]. This threshold is relevant in the epidemiology of nosocomial infections, as it is associated with an increased risk of treatment failure and the need to use second- or third-line antimicrobial agents.

For data interpretation, individual MARI values were used to calculate the average annual MARI for each of the years analysed, the average MARI per sample type, the average MAR score per department, and the average MARI per type of hospitalisation.

### 2.6. Statistical Analysis

Data collection and initial preprocessing were performed using Microsoft Excel. Statistical analyses were then conducted using the R (version 4.5.1), The R Foundation for Statistical Computing, Vienna, Austria [27], with the following packages: arsenal [28] for dataset comparison, gtsummary [29] for generating descriptive and inferential statistical tables, ggplot2 [30] for graphical data visualisation, and ggsankey [31] for constructing Sankey diagrams to illustrate relationships between categorical variables. The results were considered significant for a significance level of 0.05.

The normality of the data was assessed using the Kolmogorov–Smirnov test. Since the data did not meet the normality assumption, the non-parametric Kruskal–Wallis test was used to determine whether there are significant differences between the medians of the MARI values concerning the time factor. The graphical representation of the results was performed using boxplot diagrams, highlighting the quartiles of the analysed samples in relation to all considered factors. The association between categorical variables was determined using Pearson’s Chi-squared test, and the graphical representation was created using a 100% stacked bar chart. The relationship between the categorical variables (year, department, type of hospitalisation, and type of analysis) was illustrated through a chart that highlighted the flows of resources and quantities in relation to each bacterium tracked in our research.

Two statistical methods were applied to validate the longitudinal trends of the MARI. The Kendall correlation test was used to assess the existence of monotonic trends over time within each bacterial species. In parallel, generalised linear regression models were constructed to estimate the variation in the MARI as a function of year of isolation, hospital ward, and bacterial species. The first model assessed the independent effects of these variables, and the second model included the year × bacterial species interaction to capture trend differences between pathogens. Model performance was compared using the coefficient of determination (R^2^) and the Akaike information criterion (AIC).

## 3. Results

### 3.1. General Overview

The initial database included 4444 records for 2021, 55,081 records for 2022, 65,258 records for 2023, and 79,142 records for 2024, totalling 204,925 unique clinical cases initially included in the working set. After the extraction and standardisation process, 363 records for 2021, 256 records for 2022, 287 records for 2023, and 283 records for 2024 were used for statistical analysis, representing 1189 clinical cases included in this analysis (Table 1 and Table 2).

### 3.2. Pseudomonas Aeruginosa and Pseudomonas spp.

**Figure 1:**
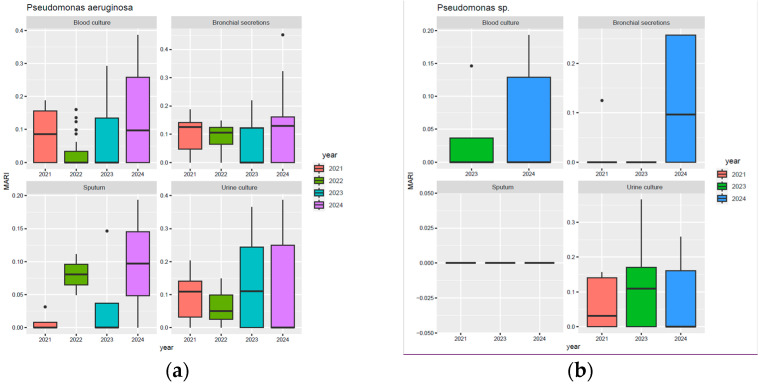
Annual evolution of the MARI in *Pseudomonas aeruginosa* and *Pseudomonas* spp. isolates collected from urine cultures, blood cultures, sputum, and bronchial secretions between 2021 and 2024; (**a**) distribution of MARI values for *P. aeruginosa* isolates; (**b**) distribution of MAR values for non-*aeruginosa Pseudomonas* spp. isolates.

For *Pseudomonas aeruginosa*, the most significant increases in the MARI were observed in urine culture samples, where values increased significantly in 2023 and remained high in 2024, also exhibiting a high degree of variation. However, an upward trend can also be observed in sputum, with a progressive increase in the median MARI from 2021 to 2024. Similarly, in blood culture, increased variability was observed in 2023 and 2024, with maximum values exceeding 0.2, compared to 2021 and 2022, when MARI values remained below 0.2 (Figure 1a). On the other hand, for *Pseudomonas* spp., MARI values remained generally low across all types of samples. However, an increase in MARI can be observed for 2024 in isolates from blood cultures, bronchial secretions, and urinary cultures, although the values remained below those observed for *P. aeruginosa* (Figure 1b).

**Figure 2:**
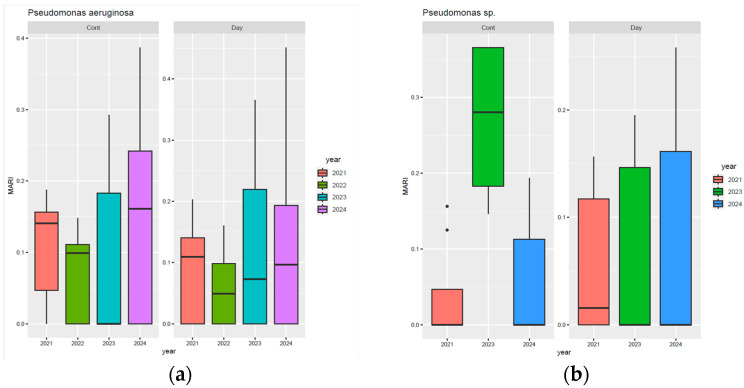
Variation in MARI values for *Pseudomonas aeruginosa* and *Pseudomonas* spp. by hospitalisation type. (**a**) *P. aeruginosa* isolates; (**b**) non-*aeruginosa Pseudomonas* spp. isolates.

Moreover, MARI values increased progressively between 2021 and 2024 in both types of hospitalisation for *Pseudomonas aeruginosa* isolates (Figure 2a). In continuous hospitalisation, a slight decrease in the median MARI value was observed, followed by a significant increase in the median value in 2024, but also marked by a wide variation and extreme values above 0.35. The same upward trend is present in day hospitalisation, with similar but slightly lower values. In contrast, regarding *Pseudomonas* non-*aeruginosa* species (Figure 2b), in continuous hospitalisation, a peak in MARI was observed in 2023, followed by a decrease in 2024, but the values for that year remain higher than in 2021. In day hospitalisation, MARI showed high variability, with a consistent increase from 2021 to 2024.

**Figure 3:**
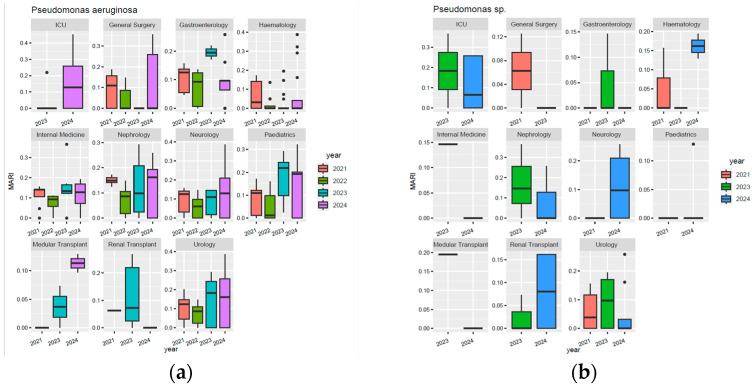
Comparative MARI values for *Pseudomonas aeruginosa* and *Pseudomonas* spp. by hospital department. (**a**) *P. aeruginosa* isolates; (**b**) *Pseudomonas* spp. isolates.

In 2024, *Pseudomonas aeruginosa* exhibited the highest MARI values in the ICU, Neurology, and Urology departments (median 0.15; extremes around 0.4) (Figure 3a). Most departments experienced a slight decrease from 2021 to 2022, followed by an increase through 2024, except Gastroenterology and Paediatrics, where a decline was noted between 2023 and 2024. For non-*aeruginosa* species, resistance generally declined in ICU, Surgery, Gastroenterology, Internal Medicine, Nephrology, Bone Marrow Transplant, and Urology. At the same time, Haematology, Neurology, and Renal Transplant displayed a marked increase in 2024 (Figure 3b).

**Figure 4:**
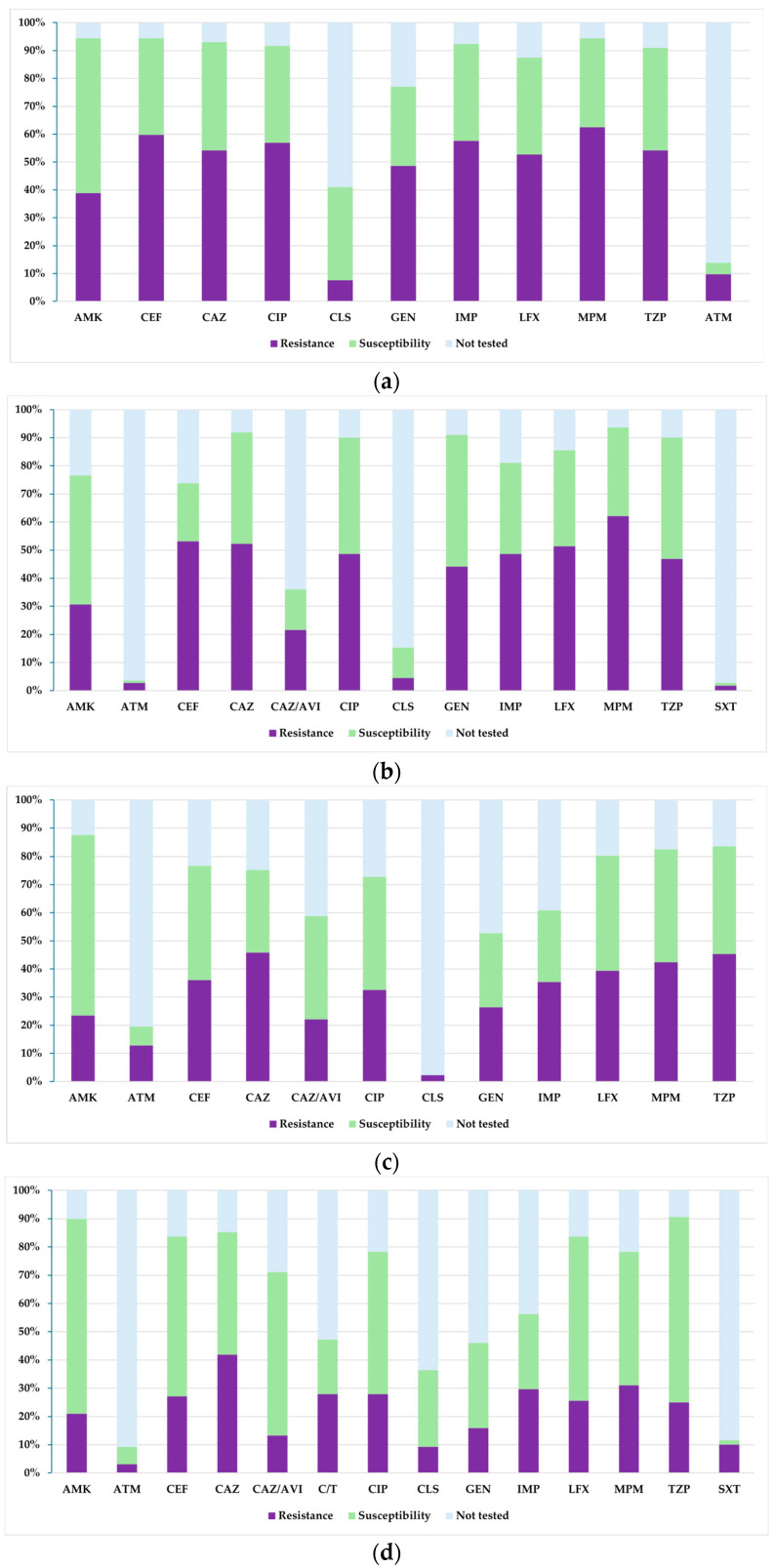
Antibiotic susceptibility profiles of *Pseudomonas* spp. isolates from 2021 to 2024: (**a**) 2021; (**b**) 2022; (**c**) 2023; (**d**) 2024. Legend: AMK—amikacin; ATM—aztreonam; CEF—cefepime; CAZ—ceftazidime; CAZ/AVI—ceftazidime–avibactam; C/T—ceftolozane–tazobactam; CIP—ciprofloxacin; CLS—colistin sulfate; GEN—gentamicin; IMP—imipenem; LFX—levofloxacin; MPM—meropenem; TZP—piperacillin–tazobactam; SXT—trimethoprim–sulfamethoxazole.

Figure 4 presents a comparative analysis of the percentages of resistance, susceptibility, and untested isolates of *Pseudomonas* spp. during the analysed period. Thus, it can be noted that for meropenem, there was a steady decrease in the proportion of resistant strains, from 63% in 2021 to 31% in 2024. Similarly, for imipenem, there was a downward trend in resistance from 58% in 2021 to 29% in 2024. Similarly, resistance to cefepime and ciprofloxacin decreased by almost half between 2021 and 2024. It should be noted that resistance to ceftazidime decreased the least, from 54% in 2021 to 42% in 2024. On the other hand, susceptibility to ceftazidime–avibactam increased four-fold during the analysed period, from 14% in 2022 to 57% in 2024. At the same time, susceptibility to piperacillin–tazobactam, levofloxacin, and cefepime also increased significantly.

### 3.3. Acinetobacter Baumannii and Acinetobacter spp.

**Figure 5:**
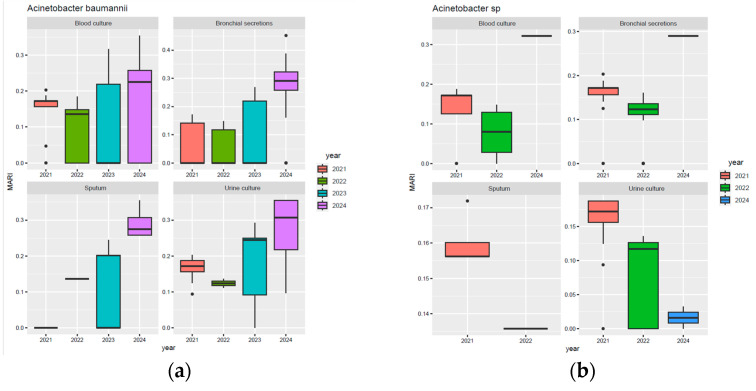
Evolution of MARI values in *Acinetobacter baumannii* and *Acinetobacter* spp. isolates stratified by biological sample. (**a**) *A. baumannii* isolates; (**b**) *Acinetobacter* spp.

Regarding *Acinetobacter baumannii* (Figure 5a), MARI values in blood cultures increased steadily from 2021 to 2024, with the highest median MARI recorded in 2024. In bronchial secretions, a progressive increase is observed, with a peak in 2024, where the median MARI was close to 0.3; however, some values reached a MARI value of 0.45. In sputum and urine cultures, the median MARI also reached its highest values in 2024, approximately 0.26 and over 0.3, respectively. Regarding *Acinetobacter* spp., a median MARI value of roughly 0.30 was observed in blood cultures and bronchial secretions in 2024 (Figure 5b).

**Figure 6:**
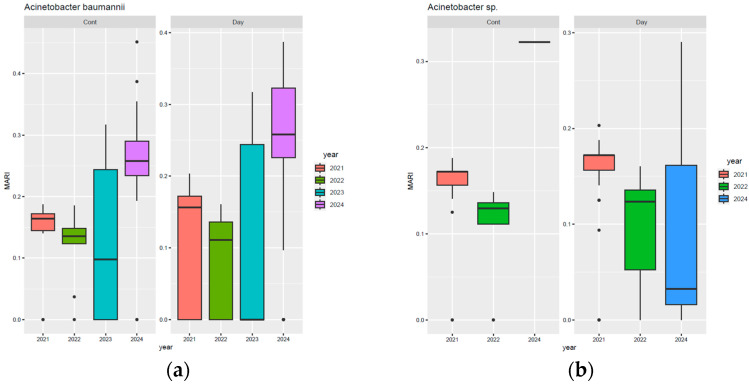
Comparison of MARI values for *Acinetobacter baumannii* and *Acinetobacter* spp. based on the type of hospitalisation. (**a**) *A. baumannii* isolates; (**b**) *Acinetobacter* spp. isolates.

For *Acinetobacter baumannii* (Figure 6a), MARI values were stable in 2021–2022 (around 0.15), increased in variability in 2023, and rose sharply in 2024 (median around 0.26; extremes up to 0.45). Day hospital isolates followed the same upward trend, with greater variability and a 2024 median of 0.26. For *Acinetobacter* spp., continuous hospitalisations showed a median peak in 2024, while day hospitalisations displayed a progressive decline from 2021 to 2024, despite higher variability in the final year (Figure 6b).

**Figure 7:**
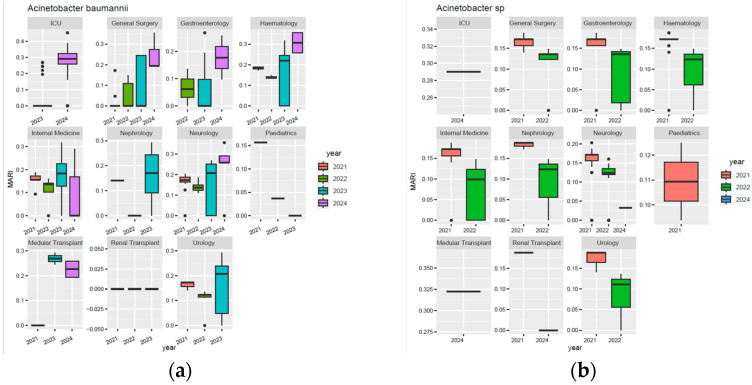
Distribution of MARI values for *Acinetobacter baumannii* and *Acinetobacter* spp. by hospital department. (**a**) *A. baumannii* isolates; (**b**) *Acinetobacter* spp. isolates.

According to Figure 7a, the highest MARI values for *Acinetobacter baumannii* were observed in the ICU, Bone Marrow Transplant, and Neurology departments, especially in 2023–2024, with ICU values rising sharply in 2024 and Neurology reaching a median of 0.25. General Surgery also showed a progressive increase, with some values exceeding 0.3 in 2024, while Haematology and Gastroenterology recorded marked rises the same year. By contrast, Paediatrics and Renal Transplantation had very low or absent values. For *Acinetobacter* spp. (Figure 7b), the highest MARI was noted in 2021 in the ICU and Bone Marrow Transplant, with stable values in Nephrology and Internal Medicine, and a decrease in Neurology by 2024 to below 0.05.

**Figure 8:**
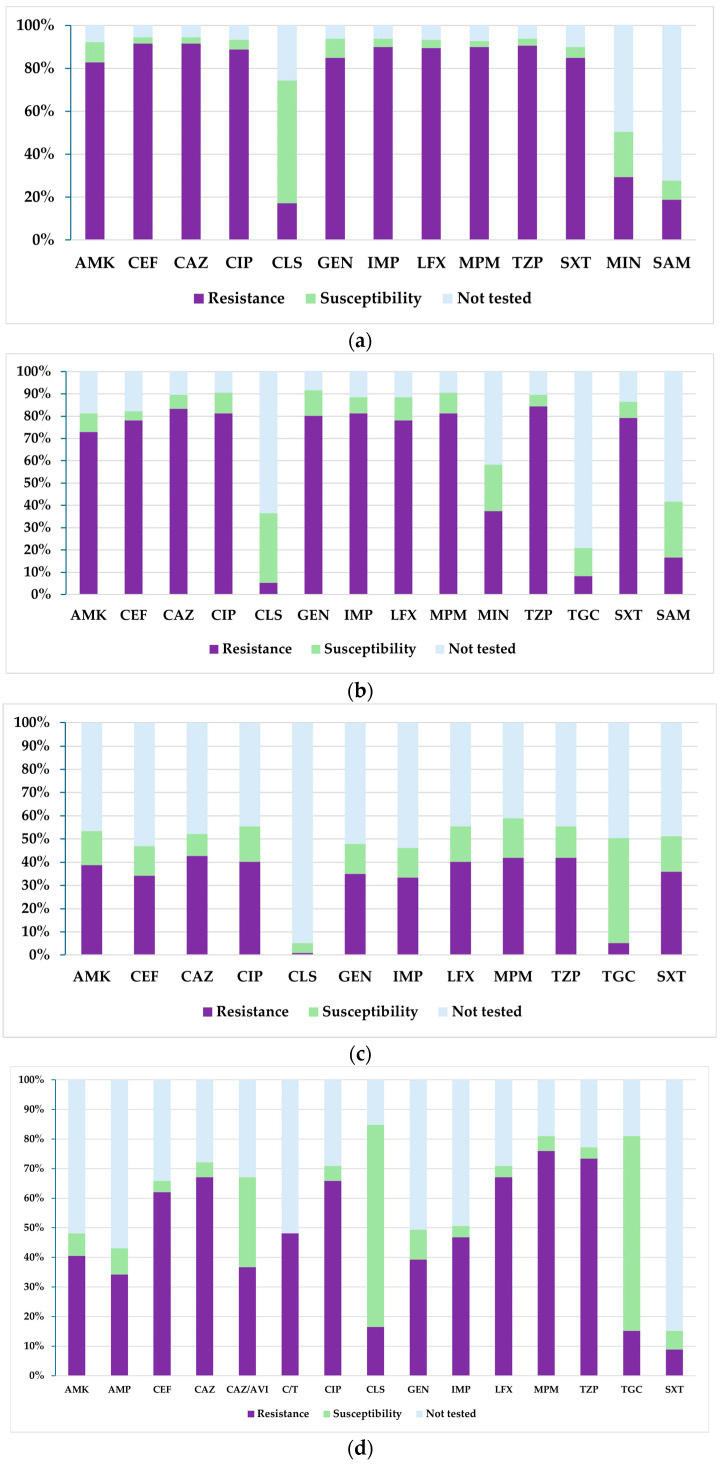
Antibiotic susceptibility profiles of *Acinetobacter* spp. isolates from 2021 to 2024: (**a**) 2021; (**b**) 2022; (**c**) 2023; (**d**) 2024. Legend: AMK—amikacin; CEF—cefepime; CAZ—ceftazidime; CIP—ciprofloxacin; CLS—colistin sulfate; GEN—gentamicin; IMP—imipenem; LFX—levofloxacin; MPM—meropenem; TZP—piperacillin–tazobactam; CXT—trimethoprim–sulfamethoxazole; MIN—minocycline; SAM—ampicillin–sulbactam; TGC—tigecycline.

Figure 8 illustrates the evolution of resistance and susceptibility to the main antibiotics tested on *Acinetobacter* spp. isolates between 2021 and 2024. A significant rebound in resistance was observed in 2024 for most of the molecules analysed. Resistance to meropenem decreased until 2023 (42%), but increased significantly in 2024, to 76%. Similarly, resistance to piperacillin–tazobactam decreased from 91% in 2021 to 42% in 2023, followed by an increase to 73% in 2024. A similar trend was observed for ceftazidime and cefepime, with increases in resistance in 2024 of 24 and 28 percentage points, respectively, compared to the previous year. For ciprofloxacin, resistance increased from 40% in 2023 to 66% in 2024. For tigecycline, tested since 2022, resistance tripled in 2024 compared to 2023, from 5% to 15%. On the other hand, there was a percentage increase in susceptibility to tigecycline, with a final value of 66%, up from 13% in 2022. Similarly, susceptibility to colistin increased significantly in 2024, from 4% in 2023 to 68%, after a progressive decline in previous years.

### 3.4. Stenotrophomonas Maltophilia

**Figure 9:**
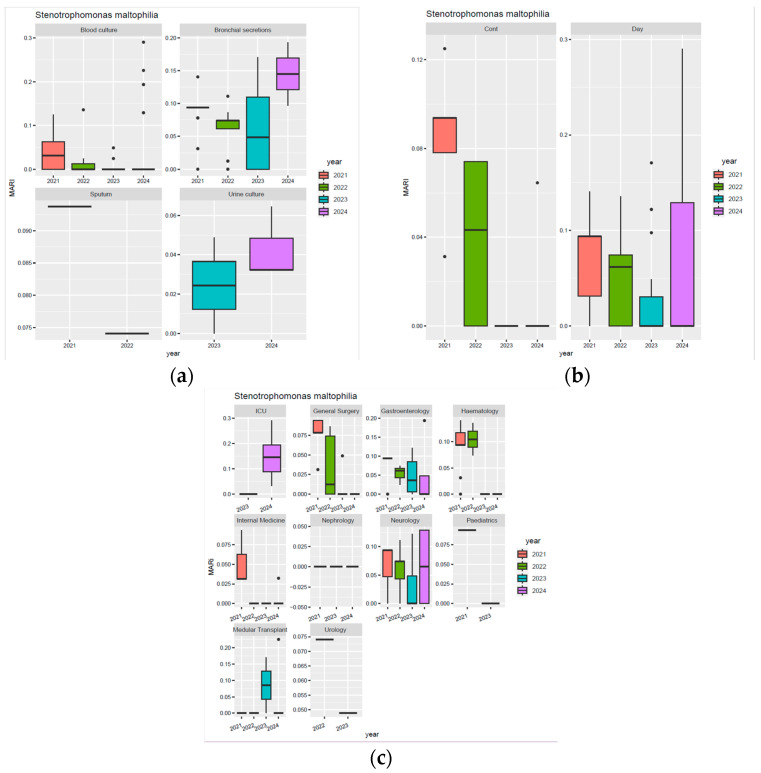
Annual variation in MARI values in *Stenotrophomonas maltophilia* isolates: (**a**) MARI values in different biological samples; (**b**) type of hospitalisation; (**c**) hospital departments.

Figure 9 illustrates the MARI values for *Stenotrophomonas maltophilia* isolates. In bronchial secretions, a slight decrease in the median MARI value was observed from 2021 to 2023. It is worth noting that in 2023, high variability was observed, with extreme values reaching nearly 0.15. In 2024, a sharp increase in the median MARI to 0.15 was observed, with extreme values close to 0.2. Similarly, in urine cultures, an upward trend was observed between 2023 and 2024, but with lower values than in bronchial secretions; extreme values in 2024 reached approximately 0.2 (Figure 9a). Moreover, in continuous hospitalisations, although a peak in the resistance of this pathogen was identified in 2021, in 2023 and 2024, a median MARI value close to 0 was observed. In contrast, day hospitalisations show a downward trend from 2021 to 2023, but a marked increase in variability and extreme values in 2024, reaching almost 0.3 (Figure 9b). Additionally, it can be observed that the departments with the highest MARI values in 2024 were ICU and Neurology. In the departments of General Surgery, Gastroenterology, Haematology, Internal Medicine, and Urology, downward trends were observed during the analysed period (Figure 9c).

**Figure 10:**
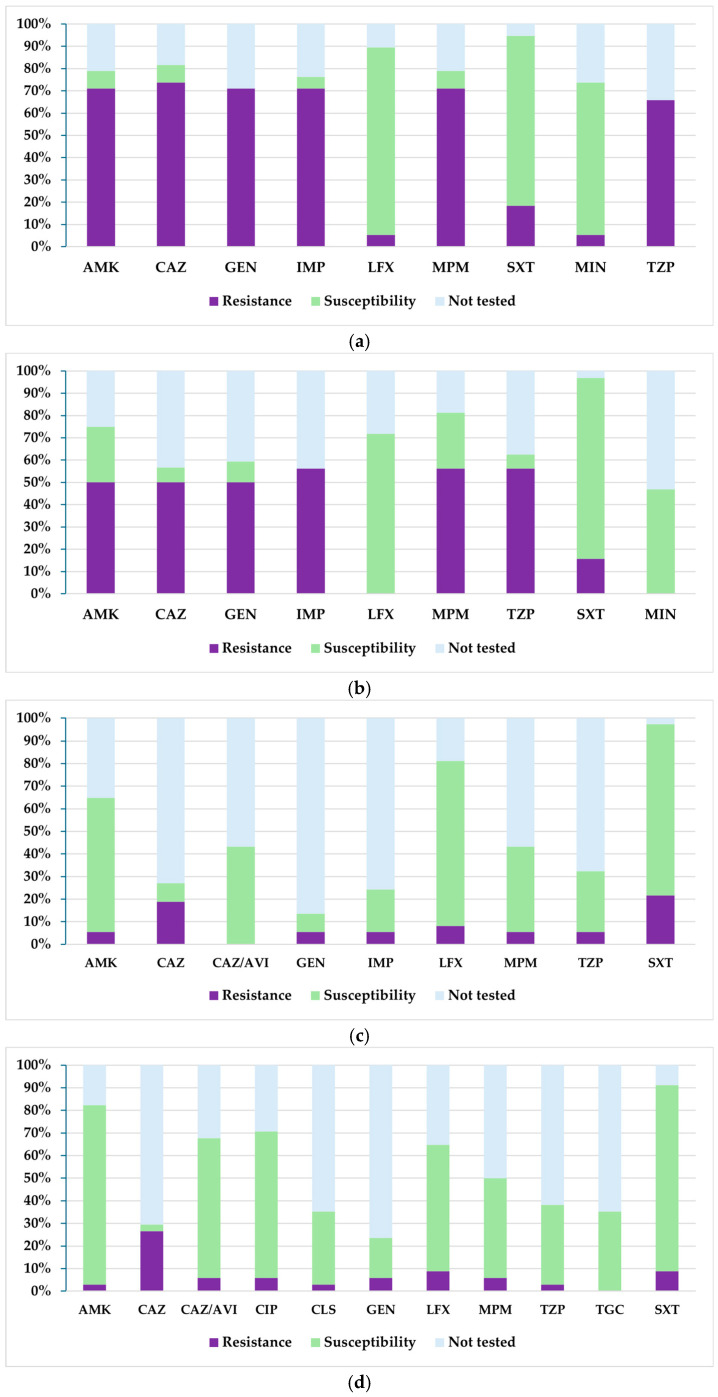
Antibiotic susceptibility profiles of *Stenotrophomonas maltophilia* isolates from 2021 to 2024: (**a**) 2021; (**b**) 2022; (**c**) 2023; (**d**) 2024. Legend: AMK—amikacin; CAZ—ceftazidime; CAZ/AVI—ceftazidime–avibactam; CIP—ciprofloxacin; CLS—colistin sulfate; GEN—gentamicin; IMP—imipenem; LFX—levofloxacin; MPM—meropenem; SXT—trimethoprim–sulfamethoxazole; MIN—minocycline; TZP—piperacillin–tazobactam; TGC—tigecycline.

Figure 10 shows the resistance and susceptibility profile for *Stenotrophomonas maltophilia* during 2021–2024. A progressive decrease in resistance to ceftazidime, gentamicin, imipenem, amikacin, meropenem, and piperacillin–tazobactam was observed, all with high initial values in 2021 and minimal values in 2023, remaining relatively constant in 2024. The most considerable reductions were observed between 2022 and 2023, particularly for gentamicin, meropenem, amikacin, and piperacillin–tazobactam, where resistance fell from over 50% to below 6%. In terms of susceptibility, trimethoprim–sulfamethoxazole maintained a favourable profile throughout the analysed period, with constant values above 75%. Increased susceptibility to levofloxacin was also observed between 2021 and 2023, but in 2024, susceptibility decreased by 20 percentage points compared to the previous year. It is important to note that susceptibility to amikacin increased steadily, from 8% in 2021 to 79% in 2024. It should also be noted that minocycline was only tested in the first two years and showed a decrease in susceptibility from 68% in 2021 to 47% in 2022.

### 3.5. Statistical Analysis of Longitudinal Trends in the MAR Index

**Figure 11:**
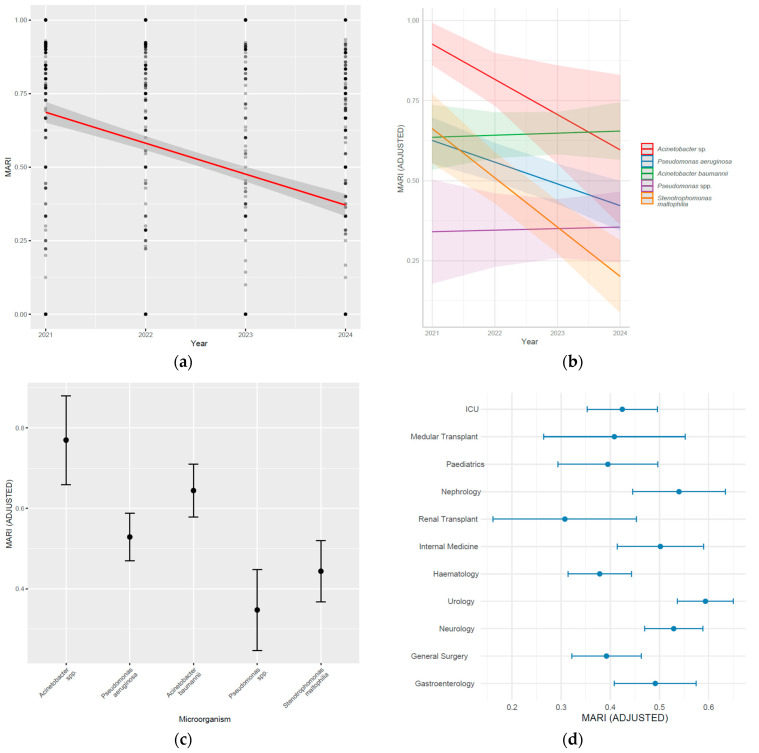
Evolution of the adjusted MARI as a function of time, pathogen, and hospital ward between 2021 and 2024. (**a**) Overall annual trend of the MARI (2021–2024), estimated on all bacterial isolates in the study; (**b**) annual evolution of the adjusted MARI for each selected bacterial species; (**c**) adjusted mean values of the MARI for each pathogen, regardless of year; (**d**) adjusted mean values of the MARI for the hospital wards.

To validate the observed longitudinal trends of the MARI, two complementary statistical methods were applied: the Kendall correlation test and generalised linear regression. Thus, Figure 11a illustrates the global evolution of the MAR index from 2021 to 2024. No clear variation in the values was observed, as they remained within the range of 0.45–0.55 throughout the entire period. The Kendall test was used to assess trend-type tendencies within each bacterial species. *Pseudomonas aeruginosa* exhibited a statistically significant decreasing trend (τ = −0.186, *p* = 6.91 × 10^−7^), followed by *Acinetobacter baumannii* (τ = −0.104, *p* = 0.0345). For *Stenotrophomonas maltophilia*, the Kendall coefficient was minimal (τ = −0.037), but with a significant p-value (*p* = 1.09 × 10^−7^), indicating low variance, possibly due to the large amount of data. For *Acinetobacter* spp. (τ = −0.093, *p* = 0.122) and *Pseudomonas* spp. (τ = −0.075, *p* = 0.395), no significant temporal trend was evident.

The linear regression model with interactions was used to estimate the variation in the MARI according to year, bacterial species, and hospital ward (Figure 11b). The comparison of the models revealed that the inclusion of Year × Bacteria interactions enhanced the model’s explanatory power (R^2^ = 0.2365 vs. 0.219) and reduced the AIC value (21,924 vs. 17,942).

Regarding independent effects of bacteria (Figure 11c), the adjusted mean MARI values were higher for *Stenotrophomonas maltophilia* (+324, 95% CI: 183–465, *p* < 0.001), *Acinetobacter* spp. (+236, CI: 41–430, *p* = 0.017), and *Pseudomonas aeruginosa* (+150, CI: 43–258, *p* = 0.006), compared to the reference bacteria (*Acinetobacter baumannii*). Statistically significant decreasing trends were observed for *Stenotrophomonas maltophilia* (coef. = −0.16, CI: −0.23–−0.09, *p* < 0.001), *Acinetobacter* spp. (coef. = −0.12, CI: −0.21–−0.02, *p* = 0.018), and *Pseudomonas aeruginosa* (coef. = −0.07, CI: −0.13–−0.02, *p* = 0.006). For *Pseudomonas* spp., the coefficient was insignificant (coef. = 0.00, CI: −0.08–0.08, *p* > 0.9).

In Figure 11d, the adjusted mean MARI values by hospital ward are shown. Compared to the reference ward (ICU), the wards with the highest adjusted values were Urology (coef. = +0.17, CI: 0.08–0.26, *p* < 0.001), Neurology (+0.10, CI: 0.02–0.19, *p* = 0.015), and Nephrology (+0.12, CI: 0.00–0.23, *p* = 0.042). The remaining wards showed statistically insignificant coefficients (*p* > 0.05).

## 4. Discussion

One of the most urgent concerns for global public health in the twenty-first century is AMR. The widespread problem is primarily attributed to the effects of excessive or irresponsible antibiotic use in various settings, including clinical care, farming, animal medicine, wartime, and the food chain. AMR, often referred to as the “Silent Pandemic”, requires immediate and effective interventions rather than being postponed until a future date [32]. Important information is provided by surveillance studies, which enable the detection of patterns of pathogen prevalence and antimicrobial resistance, as well as the identification of emergent pathogens at the national and global levels [33]. To project broad trends in resistance that may not be evident in specific hospitals but are especially important for healthcare providers to be aware of, regional, national, and international surveillance systems utilise isolated strains from hospital and reference clinical microbiology departments, as well as state public health laboratories. Multicentre AMR surveillance systems are frequently limited by the inclusion of duplicate, improperly identified, or clonal strains, which can distort surveillance data. Additionally, the lack of timely data publication in peer-reviewed scientific journals and the absence of denominator data when evaluating the significance of antibiotic resistance rates are also limitations [34]. In order to develop and improve strategies for managing antibiotic resistance and to guide clinical decisions regarding the most effective course of therapy, routine surveillance is of utmost importance [33].

The most recent ECDC country profile for Romania (2023) indicates that surveillance coverage is still limited to 13%. However, the majority of invasive cases are caused by ICU-derived isolates, particularly *Acinetobacter* spp. (62%), and *Pseudomonas aeruginosa* (46%), which are significantly higher than the EU/EEA averages. Romania continues to report significantly higher carbapenem resistance in *Acinetobacter* spp. and considerable multidrug resistance in *P. aeruginosa* in comparison to the EU/EEA mean. This is consistent with prior reports of endemic ICU transmission. It is important to note that *Stenotrophomonas maltophilia* is not included in EARS-Net surveillance, which highlights an imperative blind area in international reporting [35]. In line with the ECDC report, our results confirm that ICUs represent the main reservoir for multidrug-resistant *A. baumannii* and *P. aeruginosa*, with MARI values indicating intense selection pressure in these departments.

In Romania, healthcare-associated infections (HAIs) and AMR constitute a significantly overlooked pathology. Official reports from hospitals indicate that the prevalence rates of HAIs were merely 0.2–0.25% in 2018. A European study conducted in 2018 identified more accurate data for Romania, indicating a prevalence of 2.6%, which corresponds to approximately 100,000 registered cases annually in the country [36]. While national rates of AMR and HAIs in Romania are significantly lower than the European average, various regional studies present a contrasting perspective on the situation within the country. Szabo et al. examined HAIs, bacterial resistance, and antibiotic usage in a Romanian clinical hospital’s intensive care unit. Over one-third of 125 patients (median age 68 years, 60% male) died, with older, longer-hospitalised, and higher-antibiotic-using patients dying. The majority of patients who died were infected with *Acinetobacter baumannii*, *Klebsiella pneumoniae*, and *Pseudomonas aeruginosa*, whereas survivors were more likely to have *K. pneumoniae*, *Escherichia coli*, *Staphylococcus aureus*, *Staphylococcus hemolyticus*, and *Enterococcus faecalis*. A substantial link was found between hospitalisation time and antibiotic dosages, especially in fatal cases [37].

### 4.1. Pseudomonas spp.

*Pseudomonas aeruginosa* is the predominant pathogen associated with nosocomial infections globally [38,39]. This pathogen is a primary contributor to nosocomial infections, accounting for approximately 7.1–7.3% of all HAIs. It has an increased incidence in the ICU, where it is responsible for as many as 16.2% of infections. *P. aeruginosa* is linked to various negative health outcomes, such as ventilator-associated pneumonia (VAP), surgical site infections, urinary tract infections, and bloodstream infections (BSIs). In severe instances, including VAP and BSIs, the mortality rate may vary between 32% and 58.8% [40]. Similarly, the results obtained in our study showed a high MARI value, with extreme values reaching 0.4, particularly in blood, urine, and bronchial secretion cultures, notably in 2024. An increase in resistance of *Pseudomonas* spp. isolates, especially in blood culture samples, and the emergence of nosocomial bacteraemia with resistant strains, possibly secondary to untreated pulmonary or urinary infections, were observed. High MARI values in urine cultures may also have been caused by the inappropriate use of empirical therapy for urinary tract infections or the unjustified use of broad-spectrum antibiotics [41]. The results of our study align with those of a previous study conducted by Araya et al. in an Ethiopian hospital. A cross-sectional, five-year retrospective investigation was performed to evaluate the trend of antimicrobial resistance in *P. aeruginosa* and *A. baumannii*. A total of 1622 isolates of *A. baumannii* and *P. aeruginosa* were obtained from various clinical specimens collected between 2017 and 2021, with *P. aeruginosa* accounting for 39.4% of the total. Blood constituted the primary source of isolates at 18.3%, followed by urine at 16% and tracheal aspirate at 10.6% [42].

Regarding the type of hospitalisation, we observed higher values of antimicrobial resistance, as quantified by MARI, in cases of continuous hospitalisations. However, MARI values should not be neglected in cases of day hospitalisations either. The results obtained for continuous hospitalisations confirm the nosocomial nature of the isolates analysed, which could suggest the presence of severe pathologies in hospitalised patients and highlight the need to review empirical protocols. Furthermore, the MARI values recorded in day hospitalisations suggest a fairly widespread dissemination of *Pseudomonas* spp. in the community, a phenomenon that poses a significant threat to public health and may predict therapeutic failure in these patients, especially those with poly-comorbidities, immunocompromised patients, or those who require routine surgical interventions [43]. A study by Rajkumari et al. among trauma patients indicated that of 2269 nonduplicate *Pseudomonas* spp., the pathogen caused infections in 68% of patients whose treatment required revision but who were later discharged in good health. In 12% of cases, the infections resulted in death, while the remaining 20% of patients needed no additional interventions and improved after the removal of the offending implants/devices or following minor debridement. Additionally, it was noted that infections caused by *Pseudomonas* spp. were more prevalent in postoperative patients (84.9%) and those with prolonged hospital stays, suggesting a higher incidence of HAIs compared to preoperative patients (15.1%) [44].

Furthermore, the increased resistance observed in ICU and Transplant wards could be attributed to the high proportion of severely immunocompromised patients who are frequently exposed to broad-spectrum antimicrobials [45]. These wards often present an increased risk of selection for MDR and XDR strains and require targeted empirical treatment and rapid post-culture re-evaluation. On the other hand, increased MARI in urology wards may be caused by urinary catheters [46]. Under hypoxic and anoxic conditions, *P. aeruginosa* has been shown to proliferate slowly as unattached cell aggregates. Antibiotic recalcitrance is caused by the slow growth rates observed in the presence of a small amount of oxygen. In general, *P. aeruginosa* biofilms can be formed on abiotic surfaces, such as industrial equipment or medical implants [47].

The Infectious Diseases Society of America (IDSA) introduced the term “difficult-to-treat resistance” (DTR) to describe *P. aeruginosa* strains that exhibit a complex resistance profile, rendering them particularly challenging to manage with current antibiotics. The term DTR *P. aeruginosa* denotes *P. aeruginosa* isolates exhibiting resistance to most, if not all, commonly utilised antibiotics, such as piperacillin–tazobactam, ceftazidime, cefepime, aztreonam, ciprofloxacin, meropenem, imipenem–cilastatin, and levofloxacin [48]. In a previous study conducted at the same hospital, antibiotic consumption between 2022 and 2024 was analysed and quantified using the Defined Daily Dose (DDD). This study was conducted following the introduction of a restriction formulary as an antimicrobial stewardship measure at the Fundeni Clinical Institute at the end of 2022. A decrease in the utilisation of broad-spectrum reserve antibiotics was noted. Specifically, narrower-spectrum antibiotics have gained popularity, while reserve, broad-spectrum antibiotic prescriptions have declined. Overall, antibiotic consumption remained unchanged despite these shifts, which suggests a redistribution rather than a complete decline in use [49]. In this study, an increase in susceptibility to carbapenems and β-lactam antibiotics/β-lactamase inhibitors was observed, which could suggest the success of antimicrobial stewardship measures implemented at the institutional level. Moreover, in the same study, an increase in prescription trends for ceftazidime–avibactam was observed [49]. Similarly, in this study, we observed a more favourable susceptibility profile for this combination in 2023–2024. The increase in susceptibility suggests that ceftazidime–avibactam exhibits good activity against MDR strains and may become a preferred option for empirical treatment in severe infections; however, this does not exclude the need for local susceptibility testing.

It is important to note that, although the analysis by year revealed a decrease in resistance to most antibiotics, the increased MARI values in 2024 might suggest that the isolated strains remained largely resistant to several classes of antimicrobials simultaneously. This phenomenon could be explained by the fact that the MARI reflects the cumulative resistance of each strain, not just the proportion of susceptibility to specific agents [50]. Therefore, even if some antibiotics regain their effectiveness, the persistence of a high MARI suggests a significant therapeutic risk and requires careful monitoring and continuous adjustment of empirical protocols.

### 4.2. Acinetobacter spp.

Due to its high rate of hospital-acquired patient infections and resistance to standard antibiotic treatments, *Acinetobacter baumannii* is recognised as a problematic pathogen. Often detected in the ICUs, this microorganism causes bloodstream infections, wound infections, and life-threatening VAP, which is particularly dangerous to patients with compromised immunity [51,52]. According to research, the mortality rate for *A. baumannii*-infected ICU patients is between 45% and 60%, and it can exceed 80% when the microbes exhibit significant medication resistance. The accelerated dissemination of MDR *A. baumannii* poses a significant threat to public health [52,53]. In this direction, the carbapenem susceptibility of 18,412 *Acinetobacter* spp. isolates from 30 European countries was tested between 2013 and 2017. In Europe, the population-weighted mean percentage of *Acinetobacter* spp. that were not susceptible to carbapenem was 35.6%. At 75.5% and 71.5%, respectively, the prevalence of carbapenem-non-susceptible isolates was highest in Southern and Eastern Europe. On the other hand, the proportions of carbapenem-non-susceptible isolates in Northern and Western Europe were 2.8% and 6.3%, respectively. *Acinetobacter* spp. isolates from the ICUs had very high population-weighted mean proportions of carbapenem-non-susceptible bacteria, up to 54.0% [54]. In line with these findings, our results showed that *A. baumannii* exhibited a highly aggressive resistance profile, particularly in systemic infections, where therapeutic options are already limited. At the same time, the high MARI values in bronchial secretions and urinary cultures might be a consequence of the high adaptive capacity of *Acinetobacter* species, which form biofilms [55]. Biofilms consist of bacterial populations on biotic or abiotic surfaces, encased in an extracellular matrix, and are significant in pathogenesis, complicating treatment options. While various biological and environmental factors contribute to the formation of *A. baumannii* biofilms, glucose is the primary component. Infections caused by biofilm-forming *A. baumannii* are the predominant type associated with medical devices and present significant treatment challenges [56]. This is supported by the results presented in Figure 7, which shows that the highest MARI values were identified in the ICU, Haematology, Urology, and Bone Marrow Transplant sections. Accordingly, a prospective cross-sectional investigation, carried out in India between January and December 2023, revealed that out of 108 confirmed cases of *Acinetobacter* infection, 73 patients were male, 49 patients were over the age of 50, and 49 patients were admitted from the ICU. The predominant specimens identified were endotracheal aspirates, accounting for 36.1%, and sputum, comprising 34.2%. *Acinetobacter* species were isolated from ICU patients at a higher frequency (38%) compared to other wards [57]. Furthermore, in another investigation conducted in an Italian hospital, 89.5% of the 191 isolates were classified as MDR. In 2020, the highest number of isolates was recorded, with a 40.9% increase in blood cultures and a 62.5% increase in urine samples. In 2020 and 2022, the introduction of antimicrobial diagnostic stewardship initiatives was associated with a decrease in carbapenem resistance. Nevertheless, resistance to colistin and meropenem persisted. Effective infection prevention strategies were indicated by a 60.4% decrease in overall isolation from 2020 to 2023 [52].

In our study, high MARI values in continuous hospitalisation confirm the classic nosocomial nature of *Acinetobacter baumannii* infections, supported by the persistence of this pathogen in highly medicalised environments such as ICUs and transplant centres [58]. The expansion of community transmission, an emergent phenomenon with the potential to compromise the efficacy of empirical treatment initiated on an outpatient basis, is demonstrated by the increase in MARI observed in day hospitalisation [59]. These results underline the need to reassess empirical protocols, particularly in extra-hospital settings, and reinforce the importance of enhancing microbiological surveillance measures at the regional level.

In contrast to the previously observed downward trend, data from 2024 suggest a concerning resurgence of XDR strains of *Acinetobacter baumannii*. This poses significant issues for therapeutic management. The accumulation of complex resistance mechanisms, particularly those involving enzymatic and efflux systems, might be a primary cause of the decrease in the efficacy of first-line antimalarial molecules [60]. Under these conditions, the empirical use of carbapenems becomes unsafe, and current guidelines recommend prescribing them only in combination or based on rapid sensitivity testing [61,62]. Increasing resistance to fluoroquinolones and β-lactams limits first-line options. In this context, therapeutic options are limited to reserve agents. Colistin, a polymyxin antibiotic with concentration-dependent bactericidal activity, remains an important alternative in the treatment of severe infections, although its use is associated with an increased risk of nephrotoxicity [63,64]. Colistin administration requires careful monitoring, and combination with agents such as sulbactam or rifampicin may have a synergistic effect, contributing to increased efficacy and reduced risk of secondary resistance [65,66]. Therefore, the results underline the need to intensify antibiotic stewardship programs, optimise doses through pharmacokinetic monitoring, and consider combination therapies as a mandatory strategy in severe *Acinetobacter* infections.

### 4.3. Stenotrophomonas Maltophilia

Previously known as *Pseudomonas maltophilia* or *Xanthomonas maltophilia*, *Stenotrophomonas maltophilia* has become a significant nosocomial infection in clinical settings [67,68]. It is the cause of a variety of infectious diseases, with pneumonia and systemic infections being the most prevalent, and the mortality of hospitalised patients, particularly those who are immunocompromised, immunosuppressed, or have medical implants [69,70]. The mortality rate for nosocomial infections caused by *S. maltophilia* is significant, ranging from 12.5 to 41% for bacteraemia, 40 to 50% for respiratory diseases, and 39% for endocarditis [71]. The results of our study indicated that *S. maltophilia* had a significantly high MARI in bronchial secretion samples in 2023 and 2024. However, it is important to note that the median MARI value tripled in a single year. This phenomenon may be explained by several other reports, which have indicated different risk factors for *S. maltophilia* infection, such as immunodeficiency, chronic respiratory illness, prolonged antibiotic use, extended hospitalisation or ICU admission, and intravenous device placement [72]. In their study, Tanuma et al. reported that *S. maltophilia* was isolated from sputum with greater frequency. Nonetheless, the majority of cases involved colonisation, with instances of infection being infrequent. The authors concluded that early initiation of treatment for *S. maltophilia* infection is warranted when the pathogen is identified in sterile sites, including blood cultures and pleural fluid, or from sputum in patients with a high Sequential Organ Failure Assessment score and central venous catheter insertion [71].

One of the most unexpected results was the variance in MARI observed across different types of hospitalisation. The median MARI value in day hospitalisation decreased progressively from 2021 to 2023, when it reached an absolute value of 0, which was maintained in 2024. However, it is concerning that a significant variation is observed in day hospitalisations, particularly in 2024, when the extreme MARI values reached 0.3. This suggests a possible spread of *Stenotrophomonas maltophilia* strains in the community. Thus, the burden of HAIs may be exacerbated by the introduction of community-acquired pathogens into hospitals by day patients. Although these patients are admitted in the morning and discharged in the afternoon, usually for routine tests, they can introduce several microorganisms widespread in the community into the hospital [45]. Infection control measures can therefore be affected by the potential of these microorganisms to spread to other patients or healthcare personnel and require special attention. These observations once again underscore the need for clear regional and national protocols for monitoring nosocomial and community-acquired infections, thereby obtaining relevant data to inform therapeutic decisions [36]. According to the same line of reasoning, a study carried out by Chang et al. concluded that community-onset *S. maltophilia* infection is worthy of consideration. The authors found that a significant portion (38.6%) of 153 patients with *S. maltophilia* bloodstream infection had a community onset. Healthcare-associated infections accounted for 54.2% of *S. maltophilia* bloodstream infections, whereas community-acquired infections accounted for 45.8%. The groups with community-acquired, healthcare-associated, and hospital-acquired infections had crude mortality rates of 11.1%, 18.8%, and 60.6%, respectively [73].

Additionally, we found a high MARI in the ICU and Neurology Departments in 2024. These results are supported by previous studies. For example, Cristina et al. reported a case of *S. maltophilia* that occurred in an ICU at a hospital located in the northern region of Italy. Following an examination of the chronological and spatial course of the epidemic, it has been hypothesised that the dissemination of *S. maltophilia* could have occurred through cross-transmission during care practices [74].

According to a recent review, trimethoprim–sulfamethoxazole is the most often prescribed therapy and the only one with EUCAST breakpoints. Other drugs, including fluoroquinolones (ciprofloxacin and levofloxacin) and minocycline, have been recommended throughout the years, particularly for patients with intolerance or allergy to trimethoprim–sulfamethoxazole. Over time, ceftazidime, doxycycline, tigecycline, and moxifloxacin have been used clinically. Some less common drugs are unavailable in various countries, and intravenous therapy is usually used [75]. According to our results, trimethoprim–sulfamethoxazole remains a first-line therapeutic option, but the decrease in susceptibility to fluoroquinolones in 2024 reduces therapeutic flexibility, especially in cases of allergy or contraindications. Although the increase in susceptibility to amikacin is promising, prescribing aminoglycosides carries a risk of nephrotoxicity and requires dose adjustments and monitoring of plasma concentrations [64]. There has been a noted increase in resistance to carbapenems and β-lactams. Consequently, these molecules are not suitable for empirical therapy, and the clinical pharmacist must evaluate susceptibility profiles before commencing any broad-spectrum parenteral treatment. In addition, Banar et al. highlighted that, over time, *S. maltophilia* infections have become more common. A study of the antibiotic resistance of *S. maltophilia* before and after 2010 revealed a growing trend in resistance to some antibiotics, including ticarcillin–clavulanic acid and tigecycline. Nevertheless, trimethoprim–sulfamethoxazole is still regarded as a first-line antibiotic for treating infections caused by *S. maltophilia* [76].

### 4.4. The One Health Approach and Antimicrobial Stewardship Strategies

The One Health approach is characterised by a collaborative effort among multiple disciplines aimed at addressing the health of humans, animals, and the environment [77]. AMR is associated with each of these three components as a result of the irrational and inappropriate consumption of antimicrobials across multiple sectors, including agriculture, livestock, and human medicine [78,79]. Bacteria develop mobile genetic elements and resistance genes in response to antimicrobial selection, which may eventually spread to different bacteria of the same or other species. Bacterial acquisition of antimicrobial resistance correlates with an enhanced capacity for proliferation in animals, humans, and the environment [80].

AMR issues and prevention require surveillance and research. The One Health initiatives promote evidence-based therapies for antimicrobial resistance. Both human and non-human antimicrobial resistance, as well as prescribing patterns, must be monitored to assess the extent, trends, and health burden of resistance at the national, regional, and global levels [81,82]. Such surveillance should reveal clinically significant trends in antibiotic resistance. Surveillance should guide antimicrobial resistance education, usage policies, and stewardship initiatives. Monitoring treatments and other approaches to reduce antimicrobial resistance requires surveillance [83]. Antimicrobial use surveillance should estimate national antimicrobial consumption, using denominators such as population size to enable comparisons across nations [84].

The World Health Organisation recommends multiple interventions. Establishing strong national action plans linked with the Global Action Plan on AMR, enhancing surveillance systems to track resistance trends, and improving healthcare facility infection prevention and control are recommended [85]. To optimise antibiotic usage, the WHO recommends antimicrobial stewardship programs, quality-assured medications and diagnostics, and healthcare worker training and education [86]. Public awareness efforts, antibiotic over-the-counter regulation, and research and development for novel antimicrobials, vaccines, and diagnostics are also urgent [87]. Of note, Sadeq et al. conducted a systematic review and meta-analysis that identified 69 interventions aimed at addressing AMR and HAIs throughout Europe. Effective strategies integrated infection prevention and control measures, including hand hygiene, environmental cleaning, and isolation precautions, with antimicrobial stewardship interventions such as audit and feedback, restriction policies, and guideline implementation. Following intervention with the antimicrobial stewardship multidisciplinary team, there were significant decreases in readmissions, mortality rates, and antibiotic prescriptions. Moreover, the inclusion of a pharmacist in the Antimicrobial Stewardship Multidisciplinary Team resulted in more substantial improvements in measured outcomes compared to studies lacking pharmacist involvement [88]. Additionally, in 2018, an Australian clinic implemented a simple educational intervention for general practitioners (GPs) to improve antibiotic prescription. GPs received face-to-face training on AMS concepts, antimicrobial resistance, prescription recommendations, and microbiological testing. GP antimicrobial prescription audits revealed Australian guidelines violations, and the prescription patterns increased effectively after a basic teaching program [89]. These interventions, successfully applied in various countries, provide a practical roadmap for improving AMR management in Romania.

In simple terms, antimicrobial use monitoring should occur at the prescribing level, such as in hospitals and communities, to provide information that aids in evaluating and directing prescribing patterns [84,90]. Surveillance data must be analysed, interpreted, and communicated in an integrated manner across sectors, with reports delivered promptly to ensure their utility for relevant stakeholders. Further research is required to enhance antimicrobial stewardship, including the development of improved diagnostic tools, strategies for optimising antimicrobial prescribing and utilisation behaviours, and the advancement of more effective vaccines [91,92].

### 4.5. Key Findings, Clinical Implications, and Limitations

The MAR index serves to evaluate the risk of antimicrobial resistance (AMR) in research, highlighting regions characterised by elevated rates of antibiotic misuse. This helps in understanding the broader implications of antibiotic resistance dissemination. A MAR index value greater than 0.2 indicates a high-risk source of contamination associated with significant antibiotic use, while a value less than 0.2 signifies a source linked to low antibiotic use [93,94]. In our study, *Pseudomonas* isolates showed a median MARI value of 0.15, with extremes up to 0.40. However, other studies have reported higher MARI values, which differ depending on the geographical region. Thus, in Chad, Ahmat et al. (2023) [95] reported values ranging from 0.33 to 0.86, while in Nigeria, Adejobi et al. (2021) [96] revealed that 84.2% of isolates had a MARI between 0.50 and 1. Also, in Nigeria, Agbo et al. (2021) [97] described a MARI range between 0.27 and 0.91. Furthermore, for *Acinetobacter* species, in our study, the MARI showed median values between 0.20 and 0.30, with extremes up to 0.40. Similar and comparable results were also reported in other regions. In Kenya, Kipsang et al. (2023) [98] described MARI values ranging from 0.60 to 0.90, while in South Africa, Yew Anan et al. (2019) [99] reported lower values between 0.20 and 0.52. Likewise, in Southwest Nigeria, Adeyemi et al. (2025) [100] showed that 88% of isolates exhibited MARI values above 0.20, while in Northwest China, Meimei Hu et al. (2024) [101] reported values above 0.30, reaching up to 0.80. For *Stenotrophomonas maltophilia*, the MARI values obtained in our study ranged from a median of 0.15 to extremes of 0.30. These results are considerably lower than those reported in Mexico (2020) by Elufisan et al. [102], who described a much higher range between 0.67 and 0.90. Thus, the values obtained in our study reflect resistance profiles relevant to the Romanian context, which are mainly similar to those reported in the international literature. Nevertheless, the trend continues to be concerning and underscores the necessity of rigorous resistance profile monitoring. It is important to note that although the MARI represents a valuable quantitative tool for assessing antimicrobial selective pressure and characterising resistance profiles in a clinical context, it is relatively rarely reported in the international literature, which limits the possibilities for comparison between studies and regions.

Additionally, as shown in Figure 11, significant decreases in MARI over time were observed for *Pseudomonas aeruginosa* and *Stenotrophomonas maltophilia*, which may suggest a positive impact of antimicrobial control interventions or changes in selection pressure. However, *Acinetobacter spp.* maintain high MARI values, particularly in some wards, especially in the ICU, indicating the persistence of widely resistant strains and continued exposure to broad-spectrum antibiotics [103]. The significant differences between wards provide arguments in favour of the implementation of locally tailored antibiotic therapy policies based on the specific epidemiologic profiles of each clinical area [104,105]. In addition, high MARI values in wards such as Urology and Nephrology support the need for periodic reassessment of empiric protocols to limit the inappropriate use of carbapenems and other reserve agents. Furthermore, high MARI values are indicative of bacterial resistance that restricts treatment with last-line antibiotics. These drugs often have limited efficacy, an unfavourable safety profile, and reduced therapeutic options, increasing the risk of therapeutic failure and mortality. Their repeated use favours the emergence of additional resistance, further reducing the availability of alternatives [106].

This study has some limitations that should be mentioned. In the context of our research, the lower MARI values recorded for *Pseudomonas* spp. and *Acinetobacter* spp. during 2021–2024 do not necessarily reflect a decrease in the presence of these species in the hospital or a reduction in the number of patients infected with strains belonging to these genera. The category *Acinetobacter* spp. refers to strains that belong to the genus but have not been identified at the species level or belong to species other than *A. baumannii*, such as *A. pittii*, *A. nosocomialis*, *A. lwoffii*, *A. junii*, *A. haemolyticus*, etc. Similarly, the category *Pseudomonas* spp. encompasses all non-*aeruginosa* species, including *P. putida, P. fluorescens, P. stutzeri, P. mendocina*, etc. Several technical and methodological factors may influence variations in the observed values over the four years. Firstly, bacterial identification techniques have been continually updated over the years, and it is now possible that certain species previously classified generically as *Pseudomonas* spp. or *Acinetobacter* spp. have been identified with greater accuracy as *P. aeruginosa* or *Acinetobacter baumannii*, particularly through the use of automated laboratory systems with extensive databases. Second, isolation, susceptibility, and interpretation methods for antibiograms may vary between years, influenced by changes in laboratory protocols, CLSI/EUCAST updates, alterations in test panels, or modifications in reporting algorithms. Furthermore, variability related to the clinical context, such as the type of hospitalisation, the source of infection, and the profile of hospitalised patients, may contribute to these differences. Thus, a lower MARI value for *Pseudomonas* spp. or *Acinetobacter* spp. in a particular year does not mean that these strains have disappeared, but may, in fact, illustrate improved diagnostic accuracy and a relative increase in the proportion of strains identified and reported specifically as *Pseudomonas aeruginosa* or *Acinetobacter baumannii*.

## 5. Conclusions

This four-year research on *Acinetobacter* spp., *Pseudomonas* spp., and *Stenotrophomonas maltophilia* isolates from a Romanian reference hospital demonstrated persistently elevated resistance levels in *A. baumannii* and *P. aeruginosa*, with MARI values surpassing 0.25 in critical care units, whereas *S. maltophilia* exhibited lower yet increasingly variable resistance patterns. These findings suggest that empirical therapy for severe infections, especially in ICU and transplant patients, should be informed by current local resistance profiles, as improper antibiotic selection in these contexts can significantly affect patient outcomes. The findings indicate that multidrug-resistant non-fermenting Gram-negative bacilli continue to pose a significant risk in Romanian hospitals, highlighting the necessity for targeted infection control strategies and tailored antimicrobial stewardship initiatives. Future research must broaden surveillance efforts beyond individual centres, incorporate molecular typing to monitor clonal dissemination, and enhance national reporting systems to provide dependable, actionable data for clinical decision-making.

## Figures and Tables

**Table 1:** Evolution of the distribution by year, types of hospitalisation, departments of origin, types of biological samples, and microorganisms.

Characteristic	N = 1189 ^1^
Year	
2021	363 (31%)
2022	256 (22%)
2023	287 (24%)
2024	283 (24%)
Hospital department	
ICU	172 (14%)
General Surgery	141 (12%)
Gastroenterology	87 (7.3%)
Haematology	142 (12%)
Internal Medicine	79 (6.6%)
Nephrology	63 (5.3%)
Neurology	226 (19%)
Paediatrics	50 (4.2%)
Bone Marrow Transplantation	27 (2.3%)
Renal Transplant	25 (2.1%)
Urology	177 (15%)
Hospitalisation type	
Continuous hospitalisation	277 (23%)
Day hospitalisation	912 (77%)
Biological sample	
Blood culture	296 (25%)
Bronchial secretions	451 (38%)
Sputum	47 (4%)
Urine culture	395 (33%)
Microorganism	
*Acinetobacter baumannii*	279 (23%)
*Acinetobacter* spp.	216 (18%)
*Pseudomonas aeruginosa*	460 (39%)
*Pseudomonas* spp.	93 (7.8%)
*Stenotrophomonas maltophilia*	141 (12%)

^1^ n (%).

**Table 2:** Annual evolution of distribution by department, type of hospitalisation, type of test, and pathogen, with analysis of MARI values.

Variables	N	2021 N = 363 ^1^	2022 N = 256 ^1^	2023 N = 287 ^1^	2024 N = 283 ^1^	*p*-Value ^2^
**Hospital department**	1189					<0.001
ICU		0 (0%)	0 (0%)	54 (19%)	118 (42%)	
General Surgery		54 (15%)	51 (20%)	27 (9.4%)	9 (3.2%)	
Gastroenterology		29 (8.0%)	23 (9.0%)	18 (6.3%)	17 (6.0%)	
Haematology		53 (15%)	20 (7.8%)	46 (16%)	23 (8.1%)	
Internal Medicine		31 (8.5%)	18 (7.0%)	13 (4.5%)	17 (6.0%)	
Nephrology		13 (3.6%)	16 (6.3%)	19 (6.6%)	15 (5.3%)	
Neurology		105 (29%)	69 (27%)	27 (9.4%)	25 (8.8%)	
Paediatrics		15 (4.1%)	13 (5.1%)	8 (2.8%)	14 (4.9%)	
Bone Marrow Transplantation		5 (1.4%)	2 (0.8%)	7 (2.4%)	13 (4.6%)	
Renal Transplant		4 (1.1%)	1 (0.4%)	10 (3.5%)	10 (3.5%)	
Urology		54 (15%)	43 (17%)	58 (20%)	22 (7.8%)	
**Hospitalisation type**	1189					<0.001
Continuous hospitalisation		112 (31%)	54 (21%)	54 (19%)	57 (20%)	
Day hospitalisation		251 (69%)	202 (79%)	233 (81%)	226 (80%)	
**Biological sample**	1189					
Blood culture		48 (13%)	64 (25%)	84 (29%)	100 (35%)	
Bronchial secretions		180 (50%)	100 (39%)	81 (28%)	90 (32%)	
Sputum		13 (3.6%)	7 (2.7%)	14 (4.9%)	13 (4.6%)	
Urine culture		122 (34%)	85 (33%)	108 (38%)	80 (28%)	
**Microorganism**	1189					<0.001
*Acinetobacter baumannii*		35 (9.6%)	40 (16%)	117 (41%)	87 (31%)	
*Acinetobacter* spp.		146 (40%)	66 (26%)	0 (0%)	4 (1.4%)	
*Pseudomonas aeruginosa*		125 (34%)	118 (46%)	104 (36%)	113 (40%)	
*Pseudomonas* spp.		19 (5.2%)	0 (0%)	29 (10%)	45 (16%)	
*Stenotrophomonas maltophilia*		38 (10%)	32 (13%)	37 (13%)	34 (12%)	
**MARI**	1154	0.85 (0.63, 0.92)	0.77 (0.25, 0.92)	0.00 (0.00, 0.90)	0.45 (0.00, 0.80)	<0.001

^1^ n (%); median (Q1, Q3); ^2^ Pearson’s Chi-squared test; Kruskal–Wallis rank sum test; Bold text indicates main variable categories.

## Data Availability

The original contributions presented in this study are included in the article. Further inquiries can be directed to the corresponding author.

## References

[1] Ruggieri F., Compagne N., Antraygues K., Eveque M., Flipo M., Willand N. (2023). Antibiotics with novel mode of action as new weapons to fight antimicrobial resistance. Eur. J. Med. Chem..

[2] Hodea F.-V., Lazarescu A.-L., Grosu-Bularda A., Cretu A., Teodoreanou R.N., Lascar I., Hariga C.S. (2023). Antimicrobial Resistance of ESKAPE Pathogens in Major Burns Patients—One-Year Retrospective Study. Farmacia.

[3] Keenan K., Kiffer C.R.V., Carmo É.V.S., Corrêa J.S., de Abreu A.L., Massuda A., Gales A.C., Colombo A.L. (2025). Antimicrobial resistance burden estimates from the bottom-up: Research priorities for estimating the impact of antimicrobial resistance in Brazil. IJID Reg..

[4] Iluț P.A., Păpara C., Danescu S., Candrea E., Baican C., Vaida Ș., Baican A. (2023). Antibiotic Susceptibility and Resistance of Bacterial Pathogens in Chronic Leg Ulcers: A Retrospective Cohort Study. Farmacia.

[5] Naylor N.R., Hasso-Agopsowicz M., Kim C., Ma Y., Frost I., Abbas K., Aguilar G., Fuller N., Robotham J.V., Jit M. (2025). The global economic burden of antibiotic-resistant infections and the potential impact of bacterial vaccines: A modelling study. BMJ Glob. Health.

[6] World Health Organization Global Antimicrobial Resistance and Use Surveillance System (GLASS). https://www.who.int/initiatives/glass#:~:text=GLASS.

[7] Apetroaei M.-M., Adam-Dima I.-E., Belc N., Roming F.-I., Constantinescu F., Duță D., Macri A., Udeanu D.-I. (2024). Integrating Nutraceuticals in the One Health Framework: A Path to Holistic Health Solutions. Farmacia.

[8] Zhou Y., Frutos R., Bennis I., Wakimoto M.D. (2024). One Health governance: Theory, practice and ethics. Sci. One Health.

[9] Thakur J.S., Rana A., Kaur R., Paika R., Konreddy S., Wiktorowicz M. (2025). Situational analysis of human and agricultural health practice: One Health and antibiotic use in an indigenous village in rural Punjab, India. One Health.

[10] Wernli D., Harbarth S., Levrat N., Pittet D. (2022). A ‘whole of United Nations approach’ to tackle antimicrobial resistance? A mapping of the mandate and activities of international organisations. BMJ Glob. Health.

[11] Aboushady A.T., Manigart O., Sow A., Fuller W., Ouedraogo A.-S., Ebruke C., Babin F.-X., Gahimbare L., Sombié I., Stelling J. (2024). Surveillance of Antimicrobial Resistance in the ECOWAS Region: Setting the Scene for Critical Interventions Needed. Antibiotics.

[12] van Kessel S.A.M., Wielders C.C.H., Schoffelen A.F., Verbon A. (2025). Enhancing antimicrobial resistance surveillance and research: A systematic scoping review on the possibilities, yield and methods of data linkage studies. Antimicrob. Resist. Infect. Control.

[13] European Centre for Disease Prevention and Control (ECDC) (2018). Country Visit to Romania to Discuss Antimicrobial Resistance Issues.

[14] European Public Health Alliance (2019). In the Red Zone: Antimicrobial Resistance in Romania.

[15] Blejan I.E., Diaconu C.C., Arsene A.L., Udeanu D.I., Ghica M., Drăgănescu D., Burcea Dragomiroiu G.T.A., Rădulescu M., Maltezou H.C., Tsatsakis A.M. (2020). Antibiotic Resistance in Community-Acquired Pneumonia. A Romanian Perspective. Farmacia.

[16] Vlad M.A., Iancu L.S., Dorneanu O.S., Duhaniuc A., Pavel-Tanasa M., Tuchilus C.G. (2025). Colistin Treatment Outcomes in Gram-Negative Bacterial Infections in the Northeast of Romania: A Decade of Change Through Pandemic Challenges. Antibiotics.

[17] Hogea E., Muntean A.C., Bratosin F., Bogdan I.G., Plavitu O., Fratutu A., Oancea C., Bica M.C., Muntean D., Hrubaru I. (2024). Antibiotic Resistance Trends in Uropathogens during the COVID-19 Pandemic in Western Romania: A Cross-Sectional Study. Antibiotics.

[18] Vulcanescu D.D., Bagiu I.C., Avram C.R., Oprisoni L.A., Tanasescu S., Sorescu T., Susan R., Susan M., Sorop V.B., Diaconu M.M. (2024). Bacterial Infections, Trends, and Resistance Patterns in the Time of the COVID-19 Pandemic in Romania—A Systematic Review. Antibiotics.

[19] Luchian N., Eva L., Dabija M., Druguș D., Duceac (Covrig) M., Mitrea G., Marcu C., Popescu M., Duceac L. (2023). Health-associated infections in a hospital in the North-East region of Romania—A multidisciplinary approach. Rom. J. Oral Rehabil..

[20] Nabal Díaz S.G., Algara Robles O., García-Lechuz Moya J.M. (2022). New definitions of susceptibility categories EUCAST 2019: Clinic application. Rev. Española Quimioter..

[21] Fodor A., Abate B.A., Deák P., Fodor L., Gyenge E., Klein M.G., Koncz Z., Muvevi J., Ötvös L., Székely G. (2020). Multidrug Resistance (MDR) and Collateral Sensitivity in Bacteria, with Special Attention to Genetic and Evolutionary Aspects and to the Perspectives of Antimicrobial Peptides—A Review. Pathogens.

[22] Lin T.-L., Chang P.-H., Liu Y.-W., Lai W.-H., Chen Y.-J., Chen I.-L., Li W.-F., Wang C.-C., Lee I.-K. (2024). Gram-negative bacterial infections in surgical intensive care unit patients following abdominal surgery: High mortality associated with *Stenotrophomonas maltophilia* infection. Antimicrob. Resist. Infect. Control.

[23] Almasaudi S.B. (2018). *Acinetobacter* spp. as nosocomial pathogens: Epidemiology and resistance features. Saudi J. Biol. Sci..

[24] World Health Organization (2024). WHO Updates List of Drug-Resistant Bacteria Most Threatening to Human Health. https://www.who.int/news/item/17-05-2024-who-updates-list-of-drug-resistant-bacteria-most-threatening-to-human-health.

[25] Mir R., Salari S., Najimi M., Rashki A. (2022). Determination of frequency, multiple antibiotic resistance index and resistotype of Salmonella spp. in chicken meat collected from southeast of Iran. Vet. Med. Sci..

[26] Afunwa R.A., Ezeanyinka J., Afunwa E.C., Udeh A.S., Oli A.N., Unachukwu M. (2020). Multiple Antibiotic Resistant Index of Gram-Negative Bacteria from Bird Droppings in Two Commercial Poultries in Enugu, Nigeria. Open J. Med. Microbiol..

[27] R Core Team (2025). R: A Language and Environment for Statistical Computing.

[28] Heinzen E., Sinnwell J., Atkinson E., Gunderson T., Dougherty G., Votruba P., Lennon R., Hanson A., Goergen K., Lundt E. (2021). arsenal: An Arsenal of ‘R’ Functions for Large-Scale Statistical Summaries.

[29] Sjoberg D., Whiting K., Curry M., Lavery J., Larmarange J. (2021). Reproducible summary tables with the gtsummary package. R J..

[30] Wickham H. (2016). ggplot2.

[31] Sjoberg D. ggsankey: Make Sankey, Alluvial and Sankey Bump Plots in Ggplot.

[32] Ahmed S.K., Hussein S., Qurbani K., Ibrahim R.H., Fareeq A., Mahmood K.A., Mohamed M.G. (2024). Antimicrobial resistance: Impacts, challenges, and future prospects. J. Med. Surg. Public Health.

[33] Masterton R. (2008). The Importance and Future of Antimicrobial Surveillance Studies. Clin. Infect. Dis..

[34] Critchley I.A., Karlowsky J.A. (2004). Optimal use of antibiotic resistance surveillance systems. Clin. Microbiol. Infect..

[35] European Centre for Disease Prevention and Control (ECDC) (2023). Country Profiles for Antimicrobial Resistance and Healthcare-Associated Infections in Europe. 2023–2024.

[36] Szabó S., Feier B., Capatina D., Tertis M., Cristea C., Popa A. (2022). An Overview of Healthcare Associated Infections and Their Detection Methods Caused by Pathogen Bacteria in Romania and Europe. J. Clin. Med..

[37] Szabó S., Feier B., Mărginean A., Dumitrana A.-E., Costin S.L., Cristea C., Bolboacă S.D. (2025). Evaluation of the Bacterial Infections and Antibiotic Prescribing Practices in the Intensive Care Unit of a Clinical Hospital in Romania. Antibiotics.

[38] Saha P., Kabir R.B., Ahsan C.R., Yasmin M. (2025). Multidrug resistance of *Pseudomonas aeruginosa*: Do virulence properties impact on resistance patterns?. Front. Microbiol..

[39] Nițescu B., Muntean A.A., Pavel B., Ionescu L.-E., Necșulescu M., Pițigoi D., Talapan D., Popa M.I., Aramă V. (2024). Multimodal Research on Antibiotic Resistance of *Pseudomonas aeruginosa* Strains Isolated from Patients with Severe Burns in Romania. Farmacia.

[40] Hu M., Chua S.L. (2025). Antibiotic-Resistant *Pseudomonas aeruginosa*: Current Challenges and Emerging Alternative Therapies. Microorganisms.

[41] Rusu A., Petca A., Mareș C., Petca R.-C., Popescu R.-I., Negoiță S., Dănău R.-A., Chibelean C.B., Jinga V. (2023). Urinary Tract Infections in a Romanian Population: Antimicrobial Resistance of Uropathogens—A Multiregional Study. Farmacia.

[42] Araya S., Gebreyohannes Z., Tadlo G., Gessew G.T., Negesso A.E. (2023). Epidemiology and Multidrug Resistance of *Pseudomonas aeruginosa* and *Acinetobacter baumanni* Isolated from Clinical Samples in Ethiopia. Infect. Drug Resist..

[43] Sathe N., Beech P., Croft L., Suphioglu C., Kapat A., Athan E. (2023). *Pseudomonas aeruginosa*: Infections and novel approaches to treatment “Knowing the enemy” the threat of *Pseudomonas aeruginosa* and exploring novel approaches to treatment. Infect. Med..

[44] Rajkumari N., John N., Mathur P., Misra M. (2014). Antimicrobial resistance in *Pseudomonas* sp. causing infections in trauma patients: A 6 year experience from a south asian country. J. Glob. Infect. Dis..

[45] Blot S., Ruppé E., Harbarth S., Asehnoune K., Poulakou G., Luyt C.-E., Rello J., Klompas M., Depuydt P., Eckmann C. (2022). Healthcare-associated infections in adult intensive care unit patients: Changes in epidemiology, diagnosis, prevention and contributions of new technologies. Intensive Crit. Care Nurs..

[46] Medina-Polo J., Naber K.G., Bjerklund Johansen T.E. (2021). Healthcare-associated urinary tract infections in urology. GMS Infect. Dis..

[47] Thi M.T.T., Wibowo D., Rehm B.H.A. (2020). *Pseudomonas aeruginosa* Biofilms. Int. J. Mol. Sci..

[48] Tamma P.D., Aitken S.L., Bonomo R.A., Mathers A.J., van Duin D., Clancy C.J. (2022). Infectious Diseases Society of America 2022 Guidance on the Treatment of Extended-Spectrum β-lactamase Producing Enterobacterales (ESBL-E), Carbapenem-Resistant Enterobacterales (CRE), and *Pseudomonas aeruginosa* with Difficult-to-Treat Resistance (DTR-*P. aeruginosa*). Clin. Infect. Dis..

[49] Hîncu S., Apetroaei M.-M., Negulescu M.C., Blidaru A., Ghica M., Udeanu D.I. (2025). Implementation of an antibiotic restriction formulary and the impact on consumption in a Romanian hospital: A three-year retrospective study. Farmacia.

[50] Teshome A., Alemayehu T., Deriba W., Ayele Y. (2020). Antibiotic Resistance Profile of Bacteria Isolated from Wastewater Systems in Eastern Ethiopia. J. Environ. Public Health.

[51] Russo A., Serapide F. (2025). The Multifaceted Landscape of Healthcare-Associated Infections Caused by Carbapenem-Resistant *Acinetobacter baumannii*. Microorganisms.

[52] Foglia F., Ambrosino A., Bashir S., Finamore E., Zannella C., Donnarumma G., De Filippis A., Galdiero M. (2025). Prevalence of *Acinetobacter baumannii* Multidrug Resistance in University Hospital Environment. Antibiotics.

[53] Černiauskienė K., Vitkauskienė A. (2025). Multidrug-Resistant *Acinetobacter baumannii*: Risk Factors for Mortality in a Tertiary Care Teaching Hospital. Trop. Med. Infect. Dis..

[54] Ayobami O., Willrich N., Suwono B., Eckmanns T., Markwart R. (2020). The epidemiology of carbapenem-non-susceptible *Acinetobacter* species in Europe: Analysis of EARS-Net data from 2013 to 2017. Antimicrob. Resist. Infect. Control.

[55] Pour N.K., Dusane D.H., Dhakephalkar P.K., Zamin F.R., Zinjarde S.S., Chopade B.A. (2011). Biofilm formation by *Acinetobacter baumannii* strains isolated from urinary tract infection and urinary catheters. FEMS Immunol. Med. Microbiol..

[56] Gedefie A., Demsiss W., Belete M.A., Kassa Y., Tesfaye M., Tilahun M., Bisetegn H., Sahle Z. (2021). *Acinetobacter baumannii* Biofilm Formation and Its Role in Disease Pathogenesis: A Review. Infect. Drug Resist..

[57] Dhanapal B., Risha M., Saikumar C. (2025). Emerging Drug Resistance in *Acinetobacter* species: A Study on Isolation, Speciation, and Antimicrobial Susceptibility Patterns in a Tertiary Care Hospital. Biomed. Pharmacol. J..

[58] Cruz-López F., Martínez-Meléndez A., Villarreal-Treviño L., Morfín-Otero R., Maldonado-Garza H., Garza-González E. (2022). Contamination of healthcare environment by carbapenem-resistant *Acinetobacter baumannii*. Am. J. Med. Sci..

[59] De Oliveira D.M.P., Forde B.M., Kidd T.J., Harris P.N.A., Schembri M.A., Beatson S.A., Paterson D.L., Walker M.J. (2020). Antimicrobial Resistance in ESKAPE Pathogens. Clin. Microbiol. Rev..

[60] Belay W.Y., Getachew M., Tegegne B.A., Teffera Z.H., Dagne A., Zeleke T.K., Abebe R.B., Gedif A.A., Fenta A., Yirdaw G. (2024). Mechanism of antibacterial resistance, strategies and next-generation antimicrobials to contain antimicrobial resistance: A review. Front. Pharmacol..

[61] Zeng M., Xia J., Zong Z., Shi Y., Ni Y., Hu F., Chen Y., Zhuo C., Hu B., Lv X. (2023). Guidelines for the diagnosis, treatment, prevention and control of infections caused by carbapenem-resistant gram-negative bacilli. J. Microbiol. Immunol. Infect..

[62] Thacharodi A., Vithlani A., Hassan S., Alqahtani A., Pugazhendhi A. (2024). Carbapenem-resistant *Acinetobacter baumannii* raises global alarm for new antibiotic regimens. iScience.

[63] Sadyrbaeva-Dolgova S., García-Fumero R., Exposito-Ruiz M., Pasquau-Liaño J., Jiménez-Morales A., Hidalgo-Tenorio C. (2022). Incidence of nephrotoxicity associated with intravenous colistimethate sodium administration for the treatment of multidrug-resistant gram-negative bacterial infections. Sci. Rep..

[64] Hîncu S., Apetroaei M.-M., Ștefan G., Fâcă A.I., Arsene A.L., Mahler B., Drăgănescu D., Tăerel A.-E., Stancu E., Hîncu L. (2024). Drug–Drug Interactions in Nosocomial Infections: An Updated Review for Clinicians. Pharmaceutics.

[65] Lee J., Lee I., Lee K.-B., Lee S.S. (2025). Comparative effectiveness and safety of colistin-based versus high-dose ampicillin/sulbactam-based combination therapy for nosocomial pneumonia caused by carbapenem-resistant *Acinetobacter baumannii*. Antimicrob. Agents Chemother..

[66] Lee H.J., Bergen P.J., Bulitta J.B., Tsuji B., Forrest A., Nation R.L., Li J. (2013). Synergistic Activity of Colistin and Rifampin Combination against Multidrug-Resistant *Acinetobacter baumannii* in an In Vitro Pharmacokinetic/Pharmacodynamic Model. Antimicrob. Agents Chemother..

[67] Adegoke A.A., Stenström T.A., Okoh A.I. (2017). *Stenotrophomonas maltophilia* as an Emerging Ubiquitous Pathogen: Looking Beyond Contemporary Antibiotic Therapy. Front. Microbiol..

[68] Kanderi T., Shrimanker I., Mansoora Q., Shah K., Yumen A., Komanduri S. (2020). *Stenotrophomonas maltophilia*: An Emerging Pathogen of the Respiratory Tract. Am. J. Case Rep..

[69] Kanchanasuwan S., Rongmuang J., Siripaitoon P., Kositpantawong N., Charoenmak B., Hortiwakul T., Nwabor O.F., Chusri S. (2022). Clinical Characteristics, Outcomes, and Risk Factors for Mortality in Patients with *Stenotrophomonas maltophilia* Bacteremia. J. Clin. Med..

[70] Carbonell N., Oltra M.R., Clari M.Á. (2024). *Stenotrophomonas maltophilia*: The Landscape in Critically Ill Patients and Optimising Management Approaches. Antibiotics.

[71] Tanuma M., Sakurai T., Nakaminami H., Tanaka M. (2025). Risk factors and clinical characteristics for *Stenotrophomonas maltophilia* infection in an acute care hospital in Japan: A single-center retrospective study. J. Pharm. Health Care Sci..

[72] Lee Y.H., Lee J., Yu B., Lee W.K., Choi S.H., Park J.E., Seo H., Yoo S.S., Lee S.Y., Cha S.-I. (2023). Risk factors for mortality in intensive care unit patients with *Stenotrophomonas maltophilia* pneumonia in South Korea. Acute Crit. Care.

[73] Chang Y.-T., Lin C.-Y., Lu P.-L., Lai C.-C., Chen T.-C., Chen C.-Y., Wu D.-C., Wang T.-P., Lin C.-M., Lin W.-R. (2014). *Stenotrophomonas maltophilia* bloodstream infection: Comparison between community-onset and hospital-acquired infections. J. Microbiol. Immunol. Infect..

[74] Cristina M.L., Sartini M., Ottria G., Schinca E., Adriano G., Innocenti L., Lattuada M., Tigano S., Usiglio D., Del Puente F. (2024). *Stenotrophomonas maltophilia* Outbreak in an ICU: Investigation of Possible Routes of Transmission and Implementation of Infection Control Measures. Pathogens.

[75] Turnidge J., Gatermann S., Kahlmeter G., Cantón R., Wootton M., Giske C.G. (2025). Rationale for contemporary antimicrobial treatment of *Stenotrophomonas maltophilia*: A narrative review. CMI Commun..

[76] Banar M., Sattari-Maraji A., Bayatinejad G., Ebrahimi E., Jabalameli L., Beigverdi R., Emaneini M., Jabalameli F. (2023). Global prevalence and antibiotic resistance in clinical isolates of *Stenotrophomonas maltophilia*: A systematic review and meta-analysis. Front. Med..

[77] Franklin A.M., Weller D.L., Durso L.M., Bagley M., Davis B.C., Frye J.G., Grim C.J., Ibekwe A.M., Jahne M.A., Keely S.P. (2024). A one health approach for monitoring antimicrobial resistance: Developing a national freshwater pilot effort. Front. Water.

[78] Arnold K.E., Laing G., McMahon B.J., Fanning S., Stekel D.J., Pahl O., Coyne L., Latham S.M., McIntyre K.M. (2024). The need for One Health systems-thinking approaches to understand multiscale dissemination of antimicrobial resistance. Lancet Planet. Health.

[79] Tudor L., Pițuru M.-T., Gheorghe-Irimia R.-A., Șonea C., Ilie L.-I., Țăpăloagă D. (2023). Unveiling Non-Choleric *Vibrio* Species in Crustaceans and Aquatic Snails: A Comprehensive Study in the South and Southeastern Romania’s Hydrographic System. Farmacia.

[80] Velazquez-Meza M.E., Galarde-López M., Carrillo-Quiróz B., Alpuche-Aranda C.M. (2022). Antimicrobial resistance: One Health approach. Vet. World.

[81] Salam M.A., Al-Amin M.Y., Salam M.T., Pawar J.S., Akhter N., Rabaan A.A., Alqumber M.A.A. (2023). Antimicrobial Resistance: A Growing Serious Threat for Global Public Health. Healthcare.

[82] Ho C.S., Wong C.T.H., Aung T.T., Lakshminarayanan R., Mehta J.S., Rauz S., McNally A., Kintses B., Peacock S.J., de la Fuente-Nunez C. (2025). Antimicrobial resistance: A concise update. The Lancet Microbe.

[83] Tudor L., Pıțuru M.-T., Gheorghe-Irimia R.-A., Șonea C., Ilie L.-I., Țăpăloagă D. (2023). Optimizing Milk Production, Quality and Safety through Essential Oil Applications. Farmacia.

[84] McEwen S.A., Collignon P.J. (2018). Antimicrobial Resistance: A One Health Perspective. Microbiol. Spectr..

[85] Chua A.Q., Verma M., Hsu L.Y., Legido-Quigley H. (2021). An analysis of national action plans on antimicrobial resistance in Southeast Asia using a governance framework approach. Lancet Reg. Health West. Pac..

[86] Kakkar A.K., Shafiq N., Singh G., Ray P., Gautam V., Agarwal R., Muralidharan J., Arora P. (2020). Antimicrobial Stewardship Programs in Resource Constrained Environments: Understanding and Addressing the Need of the Systems. Front. Public Health.

[87] Sheerah H.A., Algwizani A.R., Alghamdi R.Q., Almohammadi E.L., Al-Qunaibe A.M., Dada H.M., Algarni H.S., Tunkar S.M., Altamimi A.M., Almuzaini Y.S. (2025). Strengthening global health security through antimicrobial resistance control: Insights from Saudi Arabia. J. Infect. Public Health.

[88] Sadeq A.A., Hasan S.S., AbouKhater N., Conway B.R., Abdelsalam A.E., Shamseddine J.M., Babiker Z.O.E., Nsutebu E.F., Bond S.E., Aldeyab M.A. (2022). Exploring Antimicrobial Stewardship Influential Interventions on Improving Antibiotic Utilization in Outpatient and Inpatient Settings: A Systematic Review and Meta-Analysis. Antibiotics.

[89] Neels A.J., Bloch A.E., Gwini S.M., Athan E. (2020). The effectiveness of a simple antimicrobial stewardship intervention in general practice in Australia: A pilot study. BMC Infect. Dis..

[90] Fridkin S.K., Srinivasan A. (2014). Implementing a Strategy for Monitoring Inpatient Antimicrobial Use Among Hospitals in the United States. Clin. Infect. Dis..

[91] Bankar N.J., Ugemuge S., Ambad R.S., Hawale D.V., Timilsina D.R. (2022). Implementation of Antimicrobial Stewardship in the Healthcare Setting. Cureus.

[92] Donà D., Barbieri E., Brigadoi G., Liberati C., Bosis S., Castagnola E., Colomba C., Galli L., Lancella L., Lo Vecchio A. (2025). State of the Art of Antimicrobial and Diagnostic Stewardship in Pediatric Setting. Antibiotics.

[93] Isaiah D.O., Otokunefor K., Agbagwa O.E. (2025). Multiple antibiotic resistance indexing and molecular identification of *Escherichia coli* isolated from clinical and nonclinical sources in Port Harcourt Metropolis, Nigeria. Pan Afr. Med. J..

[94] Perry J.A., Westman E.L., Wright G.D. (2014). The antibiotic resistome: What’s new?. Curr. Opin. Microbiol..

[95] Ahmat A.M., Oumar D.A., Abdoullahi H.O., Hama C., Yandai F.H., Gamougam K., Tidjani A., Aly S., Ouchemi C. (2024). Evaluation of Multi-antibiotic Resistance Index (MAR) and Molecular Characterization of *Pseudomonas aeruginosa* Isolated in Pathological Products from Chad. Microbiol. Res. J. Int..

[96] Adejobi A., Ojo O., Alaka O., Odetoyin B., Onipede A. (2021). Antibiotic resistance pattern of *Pseudomonas* spp. from patients in a tertiary hospital in South-West Nigeria. GERMS.

[97] Agbo M.C., Ezeonu I.M., Ike A.C., Ugwu C.C. (2020). MULTIDRUG-RESISTANCE PATTERNS AND DETECTION OF PstS GENE IN CLINICAL ISOLATES OF PSEUDOMONAS AERUGINOSA FROM NSUKKA, SOUTHEAST NIGERIA. Asian J. Pharm. Clin. Res..

[98] Kipsang F., Munyiva J., Menza N., Musyoki A. (2023). Carbapenem-resistant *Acinetobacter baumannii* infections: Antimicrobial resistance patterns and risk factors for acquisition in a Kenyan intensive care unit. IJID Reg..

[99] Anane A.Y., Apalata T., Vasaikar S., Okuthe G.E., Songca S. (2019). Prevalence and molecular analysis of multidrug-resistant *Acinetobacter baumannii* in the extra-hospital environment in Mthatha, South Africa. Braz. J. Infect. Dis..

[100] Adeyemi F.M., Akinlade E.A., Yusuf-Omoloye N.A., Ajigbewu O.H., Dare A.P., Wahab A.A., Oyedara O.O., Isiaka H.S., Usamat A.O. (2025). Carbapenem-resistance in *Acinetobacter baumannii*: Prevalence, antibiotic resistance profile and carbapenemase genes in clinical and hospital environmental strains. BMC Infect. Dis..

[101] Hu M., Sun H., Xu Y., Xu X. (2024). Antimicrobial susceptibility and genetic characteristics of multi-drug resistant *Acinetobacter baumannii* isolates in Northwest China. Front. Microbiol..

[102] Elufisan T.O., Cristina Rodriguez-Luna I., Oyedara O.O., Sanchez-Varela A., Bocanegra García V., Oluyide B.O., Flores-Treviño S., Angel Villalobos López M., Guo X. (2020). Antimicrobial susceptibility pattern of *Stenotrophomonas* species isolated from Mexico. Afr. Health Sci..

[103] Ture Z., Güner R., Alp E. (2023). Antimicrobial stewardship in the intensive care unit. J. Intensive Med..

[104] Ablakimova N., Rachina S., Smagulova G., Vlasenko A., Mussina A., Zhylkybekova A., Yessenzhulova A., Koshmaganbetova G.K., Iztleuov Y. (2025). Impact of complex interventions on antibacterial therapy and etiological diagnostics in community-acquired pneumonia: A 12-month pre- and post-intervention study. Front. Pharmacol..

[105] Giovannenze F., Del Vecchio P., Frondizi F., Rando E., Leanza G.M., Gross M.M., Frater A., Magrini E., Liguoro B., Sangiorgi F. (2025). Effect of an educational antimicrobial stewardship programme on antibiotic prescriptions’ appropriateness in three medical units of a large university hospital: An interrupted time series analysis. J. Hosp. Infect..

[106] Majumder M.A.A., Rahman S., Cohall D., Bharatha A., Singh K., Haque M., Gittens-St Hilaire M. (2020). Antimicrobial Stewardship: Fighting Antimicrobial Resistance and Protecting Global Public Health. Infect. Drug Resist..

